# Intraskeletal histovariability, allometric growth patterns, and their functional implications in bird-like dinosaurs

**DOI:** 10.1038/s41598-017-18218-9

**Published:** 2018-01-10

**Authors:** Edina Prondvai, Pascal Godefroit, Dominique Adriaens, Dong-Yu Hu

**Affiliations:** 1 Evolutionary Morphology of Vertebrates, Department of Biology, Ghent University, Ghent, Belgium; 2 Royal Belgian Institute of Natural Sciences, Directorate ‘Earth and History of Life’, Brussels, Belgium; 3 Paleontological Institute, Shenyang Normal University, Key Laboratory for Evolution of Past Life in Northeast Asia, Ministry of Land and Resources, Shenyang, China

## Abstract

With their elongated forelimbs and variable aerial skills, paravian dinosaurs, a clade also comprising modern birds, are in the hotspot of vertebrate evolutionary research. Inferences on the early evolution of flight largely rely on bone and feather morphology, while osteohistological traits are usually studied to explore life-history characteristics. By sampling and comparing multiple homologous fore- and hind limb elements, we integrate for the first time qualitative and quantitative osteohistological approaches to get insight into the intraskeletal growth dynamics and their functional implications in five paravian dinosaur taxa, *Anchiornis*, *Aurornis*, *Eosinopteryx*, *Serikornis*, and *Jeholornis*. Our qualitative assessment implies a considerable diversity in allometric/isometric growth patterns among these paravians. Quantitative analyses show that neither taxa nor homologous elements have characteristic histology, and that ontogenetic stage, element size and the newly introduced relative element precocity only partially explain the diaphyseal histovariability. Still, *Jeholornis*, the only avialan studied here, is histologically distinct from all other specimens in the multivariate visualizations raising the hypothesis that its bone tissue characteristics may be related to its superior aerial capabilities compared to the non-avialan paravians. Our results warrant further research on the osteohistological correlates of flight and developmental strategies in birds and bird-like dinosaurs.

## Introduction

Studying bone histology of extinct paravian dinosaurs (all dinosaurs closer to modern birds than to *Oviraptor*^1^) is a powerful tool to infer the biology of these feathered theropods and to better understand the early evolution of birds. Previous studies focusing on ontogenetic stage, growth rate, age, and sexual maturation in dinosaur-bird transitional forms either based their conclusions on the histology of a single element, usually the femur^2–4^, or multiple, but often non-homologous bones were investigated across different studies^5–10^ rendering interspecific comparisons difficult. As osteohistological traits vary extensively among different individuals as well as within the skeleton^11–13^, single-element analyses and comparison of non-homologous bones may lead to different interpretations on the overall growth patterns depending on the elements studied. By contrast, assessing the extent of intraskeletal histovariability gives a more complete picture on ontogenetic stages and growth trajectories, and provides new insights into allometric skeletal growth dynamics and potentially locomotor aspects. Limb morphometrics combined with growth dynamics-related osteohistological traits have been used to infer functional shifts among different elements during the ontogeny in *Psittacosaurus*^14^. However, up to now, no attempt was made for applying this approach to explore the interelemental dynamics of skeletal development in paravian dinosaurs.

Apart from lineages with secondarily reduced forelimbs, paravian dinosaurs are characterized by disproportionately long and robust arms compared with other theropods. Contrasting with other bipedal dinosaurs, the length of the humerus, radius and ulna in these paravians is comparable with, or may even exceed, that of the femur, and the total forelimb to hind limb length ratio is close to or exceeds one^15–18^. This implies heterochronic allometry with the fore- and hind limb elements having differential growth rates in different phases of ontogeny, which may also reflect locomotor aspects of the precocial – altricial developmental spectrum of ontogenetic strategies^19–21^. The precocial – altricial concept, referring to the high – low degree of functional maturity at hatching (or birth) coupled with low – high postnatal growth rates^19–22^, is usually interpreted at the level of the individual, but can also characterize different anatomical units within a developing organism. Altricially developing units have higher postnatal growth rates and relatively later functional maturation than precocial units, as also seen in the disparate growth trajectories of the fore- and hind limbs in some bird species^23–26^. In many extant birds, like anseriforms, forelimb development is relatively altricial with small size at hatching, delayed functional maturation, but higher postnatal growth rates as compared to the precocial hind limbs, which are large and functionally mature enough to perform bipedal locomotion upon hatching^21,23,24,26–29^. By contrast, phasianids hatch with comparatively well-developed wings and fledge early during ontogeny^30,31^, while the hypermorphosed (“superprecocial”) hatchlings of megapod birds have functional wings^22^ that even outperform the wings of adults^32^. Thus, precocial units have a relatively earlier growth burst and hence reach functional size and morphology earlier during ontogeny than altricial units. This diversity in developmental trajectories of bird limbs is the result of complex trade-offs between wing and leg development, combined with the balancing effect of different degrees of cooperative wing-leg usage depending on the locomotor strategy and ecology of the species^31,33^. As relative timing and rates of growth are tightly linked with these trajectories, investigating the bone tissue that also reflects relative growth differences among skeletal elements may be informative of the relative precocity level of the limbs.

Paravian theropods were bipedal animals, and the skeleton of the perinatal embryos and hatchlings known from a few fossil taxa was extensively ossified^34–38^. Thus, bipedal locomotion was likely prevalent relatively early during their posthatching ontogeny, suggesting a precocial hind limb development. Functions of the long forelimb, such as grasping, climbing, or aerial manoeuvres^39,40^, and their ontogenetic development, however, remain largely unclear, although superprecocial flight ability of hatchlings with well-ossified forelimbs and early onset of slow growth evidenced by bone histology has been suggested for enantiornithine birds^34,37,41^. The relatively large size of the humerus and antebrachial bones in paravians suggests that these bones had growth rates similar to or higher than the femur at some point during development. This crucial period of high growth rate in the forelimbs, however, may or may not be recorded in the bone tissue depending on the growth strategy and the ontogenetic stage of the animal at the time of its death. Thus, if cortical osteohistology implies a considerably lower growth rate in these forelimb elements than in the femur throughout the recorded posthatching period, it can be hypothesized that 1) large forelimb size was achieved by positive allometric growth during embryonic and/or early posthatching development with no bone record due to resorption during medullary cavity expansion; and 2) large forelimb size attained early in ontogeny implies early functional maturation; i.e. relatively precocial development. By contrast, if the preserved cortex of the forelimb bones shows histological characteristics of faster growth compared to the similarly sized femur, it suggests 1) a later ontogenetic onset of high growth rate phase in the forelimbs relatively to the femur; and hence 2) a relatively altricial forelimb development.

By sampling multiple homologous limb elements, this study aims to explore and quantify intraskeletal histovariability in four Late Jurassic non-avialan paravian dinosaurs, *Anchiornis*, *Aurornis*, *Eosinopteryx* and *Serikornis*, and in the Early-Cretaceous avialan *Jeholornis*, all from Liaoning Province, China. Qualitative and multivariate quantitative osteohistological diversity between and within the five skeletons is assessed and analysed in the context of taxonomy, ontogeny, allometric growth patterns, and individual element attributes, such as size, homology, and the newly introduced relative precocity of the bones within the skeleton. This study represents the most comprehensive and detailed bone histological analysis of dinosaur-bird transitional forms so far, and is the first osteohistology-based attempt to get insight into the allometric limb bone development and raise hypotheses about its potential functional significance in these evolutionarily important taxa.

*Institutional abbreviations* – IVPP, Institute of Vertebrate Paleontology and Paleoanthropology, Beijing; China; LPM/PMOL, Paleontological Museum of Liaoning, Shenyang, P.R. China; STM, Tianyu Natural History Museum, Shandong, China; TTU, Texas Tech University, Texas, USA; YFGP, Yizhou Fossil and Geology Park, Yixian, P.R. China.

## Methods

### Materials and thin-sectioning

For this osteohistological study, the following specimens were sampled: *Eosinopteryx brevipenna* YFGP – T5197^16^, *Serikornis sungei* PMOL-AB00200^42^, *Anchiornis huxleyi* YFGP – T5199^43^, *Aurornis xui* YFGP – T5198^17^ and *Jeholornis curvipes* YFGP – yb2^18^. Small samples of the humerus, antebrachial bones, metacarpals, first phalanx of manual digit I (D1P1), and femur of each specimen were taken during the preparation-restoration phases. The furcula was also sampled in *Anchiornis*, *Aurornis* and *Eosinopteryx* but only used qualitatively as an additional reference point for ontogenetic and skeletochronological assessments (Table 1, Supplementary Figs S1–S3, Supplementary Table S1).

**Table 1:** Sampled specimens and elements with left (L) or right (R) side indicated.

	Furcula	Humerus	Radius	Ulna	McII	McIII	D1P1	Femur
*Eosinopteryx brevipenna* YFGP – T5197	+	L	R	R	L	−	L	L
*Serikornis sungei* PMOL-AB00200	−	R	L	L	L	L	L	L
*Anchiornis huxleyi* YFGP – T5199	+^†^	L	L	R	L	L	L	L
*Aurornis xui* YFGP – T5198	+	R	R	R	R	R	R	R
*Jeholornis curvipes* YFGP – YB2	−	R	R	L	L^†^	L^†^	L^†^	R

^†^Denotes elements that have gone through restoration of unknown origin before sampling. Abbreviations: D1P1, first phalanx of manual digit I; Mc II-III, metacarpals II and III.

Complete cross sections of few millimetre long diaphyseal bone segments were carefully removed mostly from the vicinity of already broken parts with a Dremel compact saw, wherever possible at the mid diaphysis. Sampled regions in each specimen have been restored using Axson SC258 low density epoxy paste. Bone samples were embedded in epoxy-resin, mounted on glass slides and cut with a high precision saw (Isomet 1000, Buehler) to ~350 µm thickness. Thereafter, sections were manually ground down to 80–60 µm thickness on a series of silicon carbide powder of 240–400–600–800 grit sizes. Reaching the desired thickness and visual effect, sections were covered by glass cover slip. Finished sections were investigated under polarized light microscope (Nikon ECLIPSE LV100 POL), photographed with QImaging MP5.0 digital microscope camera and the images acquired with Image-Pro Insight 8.0 software.

### Qualitative assessment of ontogenetic stages

Ontogenetic stages are regarded here in the context of individual growth trajectories from juveniles to fully grown adults, as reflected in the degree of osteohistological maturity generalized over all sampled bones. Assessing the specimens’ ontogenetic stage is based on the combination of osteohistological characters that change over time and indicate the dynamics of diametric bone growth and maturation process^44–51^.

Juveniles are defined as actively growing specimens without osteohistological indicators of decreasing growth rate. Early juveniles show high cortical porosity (large primary vascular spaces) and uniform vascular density throughout the dominantly primary diaphyseal cortex without lines of arrested growth (LAGs) or other growth marks that would refer to periodic (usually annual) cessation or slow-down of growth. The periosteal surface can be abundantly perforated by vascular canals indicating active incorporation of periosteal vasculature into the growing bone by diametric osteogenesis. In fast growing elements, the relative amount of cortical woven bone forming a de novo scaffold around vascular canals is higher than in later ontogenetic stages. Combination of woven scaffold with centripetal infilling of vascular spaces by lamellar or non-lamellar parallel-fibred bone forming primary osteons results in a fibrolamellar complex (FLC, *sensu* Prondvai *et al*.^52^). Woven bone is distinguished by the irregular spatial arrangement and morphology of its osteocytic lacunocanalicular system combined with its random (large-scale isotropic) optical behaviour under cross polarized light, as opposed to the spatially aligned lacunocanalicular network and uniform optical pattern seen in parallel-fibred bone^50^. In the bones of late-juveniles growth marks may appear and porosity is relatively lower due to the more complete infilling, i.e. maturation of primary osteons which also results in a proportional decrease of woven to parallel-fibred bone.

Subadults are characterized by a notable decrease in diametric bone growth rate inferred from the decrease in vascularity and potentially closer spacing of growth marks towards the periosteal surface, but still continuing, albeit slower, growth evidenced by the persisting vascularity in the outermost cortex.

Adults are fully grown, skeletally mature individuals with bones showing complete cessation of, or only minimal, accretional diametric growth with avascular outermost cortex and often densely accumulated growth lines or lamellae referred to as external fundamental system (EFS) or outer circumferential layer (OCL), respectively^51,53^. As microdamage repair, calcium metabolism and biomechanical adaptations in the bone require secondary remodelling, which accumulates with age^54–56^, the listed primary features combined with extensive remodelling with several generations of secondary osteons often imply senescence.

Although generally considered to form annually, and hence to be informative of the specimen’s age^57–61^, LAGs and other growth mark counts were used in this study only in a qualitative referential manner because 1) the extensive medullary cavity expansion maintaining cortex thinness, and the frequently considerable remodelling in the long bones of these paravian dinosaurs result in an especially incomplete primary growth record; 2) all bones are severely crushed and several miss large cortical areas for restoring circumferential parameters; 3) the usually indistinct nature of growth marks in the studied specimens makes counting unreliable for quantitative purposes; and 4) other uncertainties related to LAG development within the cortex as well as in EFS/OCL^46,59,62–65^ also add to the high degree of inaccuracy in estimating total cyclical growth mark number.

### Qualitative and semi-quantitative assessment of relative diametric bone growth rates

Relative growth dynamics of different elements within the skeleton were assessed qualitatively by examining osteohistological characters considered to indicate diametric bone growth rates^45,52,66–68^. As ontogenetic assessment is also based on differences in bone growth dynamics, these characters largely correspond with those used for ontogenetic assignments. However, whereas inferred growth dynamics was generalized over the entire skeleton for the specimens’ ontogenetic categorization, interelement diversity in diametric bone growth rate is evaluated by comparing the inferred growth trajectories of each sampled element within the skeleton.

Accordingly, the primary diaphyseal cortex of faster growing bones shows more ‘juvenile’ osteohistological characteristics than seen in the relatively slower growing bones of the skeleton. Because all bones within a skeleton have the same age, potential differences in these traits among different elements are considered to faithfully reflect patterns of intraskeletal growth dynamics.

In addition, we established a semi-quantitative method for comparing relative bone growth dynamics by taking primary vascular profiles in the cortex, excluding the inner circumferential layer (ICL), of each element along several radial transect lines, wherever the original periosteal bone surface was preserved. Transects were placed away from each other by about 0.5–1 times of the total cortex thickness. Radial extent of each vascular canal that these transects passed through was registered on the lines along the cortex thickness standardized to 100%. Thereafter, the standardized cortex thickness was divided up to five equal segments (20%), and the number of primary vascular canals situated in each segment was visualized in a histogram to approximate the radial distribution pattern of vascularity. Distance between transects and cortex thickness division were practical arbitrary settings of sampling adjusted to these paravian bone samples to compromise between time investment and the amount of valuable information attained. The extent of secondary remodelling was also registered and their obscuring effect on original primary vascular density allowed for in the qualitative interpretation of these distribution patterns (see Supplementary Fig. S4).

### Precocity ranks of elements

Given the trade-off between growth rate and functional maturity of tissues^25,30,69–71^, a precocial musculoskeletal performance is expected to be in conflict with fast growth of the tissues involved, as also evidenced by the lower postnatal growth rates of precocial hind limbs compared to altricial forelimbs in several birds^23–26^. As locomotion-related biomechanical loads are frequently associated with osteonal development and secondary bone tissues^45,54–56,72^, relatively early growth burst combined with biomechanics-related osteohistological features likely indicate relatively precocially developing elements. Thus, we combined the listed osteohistological indicators of growth rate with osteonal development, extent of remodelling and ICL thickness, to introduce what we call ‘precocity ranks’, which aim to reflect the relative intraskeletal order of functional maturation and growth of limb bones. Precocity ranks were assigned to each bone within the skeleton following fractional ranking rules (Table 2). Bones showing the slowest growth, well-developed osteons, relatively thick ICL and high degree of secondary remodelling were considered the most ‘precocial’ and got the lowest rank in the skeleton, whereas the highest rank was assigned to the fastest growing ‘altricial’ bones with the least developed osteons and ICL, and the lowest degree of remodelling.

**Table 2:** Intraskeletal precocity ranks assigned to each skeletal element based on qualitative evaluation of histological characters without hand bones and without *Jeholornis*.

	Without hand bones					Without *Jeholornis*			
	Eos	Ser	Anch	Aur	Jeh	Eos	Ser	Anch	Aur
Humerus	4	3	4	1.5	4	6	5.5	7	4.5
Radius	1.5	2	2	3.5	2	2.5	4	2	6.5
Ulna	1.5	1	3	3.5	2	2.5	3	5	6.5
Mc II	—	—	—	—	—	4	1.5	5	2
Mc III	—	—	—	—	—	—	1.5	5	2
D1P1	—	—	—	—	—	1	5.5	1	2
Femur	3	4	2	1.5	2	5	7	3	4.5

Abbreviations: Eos, *Eosinopteryx*; Ser, *Serikornis*; Anch, *Anchiornis*; Aur, *Aurornis*; Jeh, *Jeholornis*. Anatomical abbreviations as in Table 1.

These qualitatively assessed intraskeletal element ranks represent ordered relative indices of potentially function-related growth allometries. As homologous bones were sampled in all specimens in this study, the interelemental distribution of ranks could be examined across skeletons to compare their relative developmental patterns. However, these precocity ranks are not intended to inform about osteohistological correlates of effective precocial vs. altricial limb usage, for which comparative osteohistological data on extant animals is too scarce.

Because relative precocity of elements is embedded in the framework of individual development, ontogeny and precocity ranks share the common conceptual background of bone growth and maturation processes. However, whereas the individual’s ontogenetic stage is interpreted in the context of overall unidirectional bone growth, element precocity ranks aim to capture the multidirectional effects of relative allocation pattern and trade-offs between growth and functional maturation in different bones. For instance, secondary remodelling does not contribute to bone growth but rather represents an important indicator of biomechanics and other physiological processes that may be in allocation conflict with growth. Hence, precocity ranks are qualitative abstractions of these multidirectional processes in the network of physiological and functional trade-offs shaping the bone tissue. The complex interplay between these developmental processes is expected to largely account for the osteohistological variability.

### Quantitative analysis

For numerical analysis of histovariability, we quantified in each element histological features indicative of relative growth rate and/or functional demands as defined above, as well as traits that can readily be measured but are of debated functional importance, such as vascular canal orientation. All original measurements of the dependent variables were converted into percentages to standardize them for absolute size. Sampling location relative to the total length of the bone measured from the proximal epiphysis was an explanatory variable also included in the analyses (Table 3, Supplementary Table S2). Histomorphometric measurements were performed on the thin section pictures in ImageJ, and the data analysed in R 3.3.3 and PAST3 free statistical softwares.

**Table 3:** Measured and derived morphometric and histomorphometric numeric variables used in multivariate analyses.

Variable	Method of calculation	Dependent (D)/independent (ID) variable	Number code in Fig. 3
ICL thickness %	Mean ICL thickness as % of total mean cortex thickness	D	1
Primary bone area %	Primary bone area as % of total section area	D	2
Secondary bone area %	Secondary bone area as % of total section area	D	3
Primary vascular density	Number of primary canals/mm^2^ calculated only for primary bone area	D	4
Primary longitudinal vascular canal %	Number of longitudinal canals as % of all primary canals (following canal orientation categories of Cubo *et al*.^116^)	D	5
Primary circumferential vascular canal %	Number of circumferential canals as % of all primary canals (following canal orientation categories of Cubo *et al*.^116^)	D	6
Primary oblique vascular canal %	Number of oblique canals as % of all primary canals (following canal orientation categories of Cubo *et al*.^116^)	D	7
Primary radial vascular canal %	Number of radial canals as % of all primary canals (following canal orientation categories of Cubo *et al*.^116^)	D	8
Primary irregular vascular canal %	Number of irregular (branching or winding) canals as % of all primary canals	D	9
Primary vascular area %	Total primary vascular area (porosity) as % of total primary bone area	D	10
Primary vascular diameter %	Shortest diameter of primary longitudinal canal as % of total cortex thickness	D	11
Woven bone %	Woven bone area as % of total primary bone area	D	12
EFS thickness %	Mean EFS thickness as % of total cortex thickness	D	13
Element length	Maximum measured length (or mean of both sides) of element (cm)	ID	—
Cortex thickness	Mean cortex thickness (µm)	ID	—
Mean vascular area	Mean area of primary canals (µm^2^)	ID	—
Sampling location	Distance from proximal epiphysis as % of total element length	ID	—

To explore the distribution of skeletal elements in the histomorphospace, the acquired data were analysed by principal component analysis (PCA), non-metric multidimensional scaling (NMDS), and cluster analysis. Being based on different approaches, these methods complement and corroborate each other’s results (see Supplementary Information). Taxonomic assignment of the specimen, and the homologous nature, length, cortex thickness, and sampling location of the elements were considered as potential explanatory factors for the multivariate distribution patterns. Significance of grouping factors (taxonomy and element homology) were tested by non-parametric multivariate analysis of variance (PERMANOVA), whereas the effect of numeric factors (bone length, cortex thickness, sampling location) were assessed by correlation analyses on PCA and NMDS scores. The relative importance of the numeric factors were tested by variation partitioning along with the appropriate post-hoc tests on PCA and NMDS scores.

In order to test the correspondence between our qualitative osteohistological assessments and the quantitative distribution pattern, we used the inferred ontogenetic stage of the specimens (grouping factor) and precocity ranks of elements (numeric factor) as explanatory variables and performed the same analyses as described above for the non-histology-based explanatory factors. This way, the relative impact of both qualitative osteohistological categories, the widely used ontogenetic stages and the novel precocity ranks, could also be tested against that of the non-histology-based explanatory factors.

All data used in these analyses are available in the current study and its Supplementary Information. For more details on applied R packages, scripts, functions and analyses, see Supplementary Information.

## Results

### Ontogenetic stages and estimated ages

**Figure 1:**
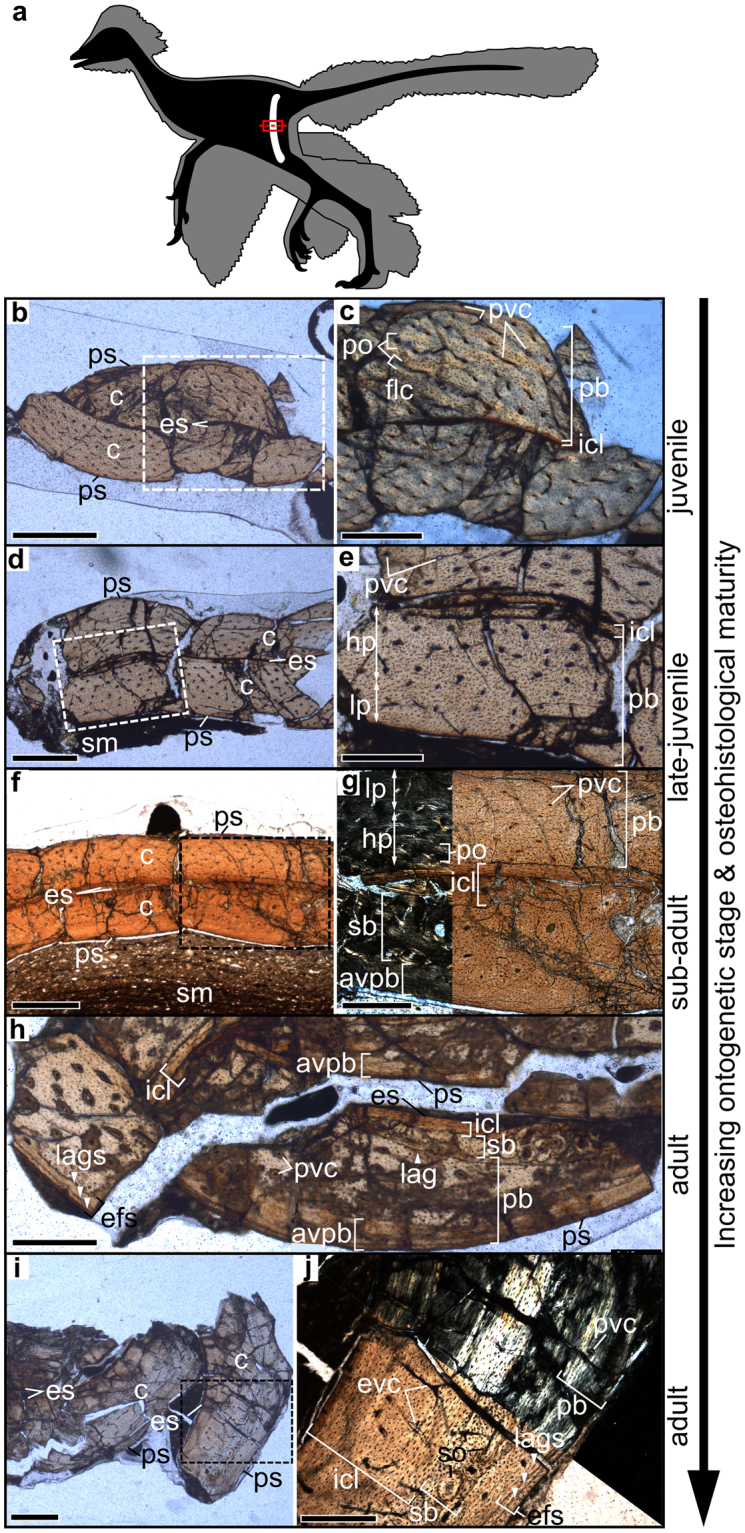
Osteohistologically defined ontogenetic stages exemplified on the femora of the studied paravian dinosaurs. (**a**) General bauplan of a bird-like dinosaur (based on *Anchiornis*^69^ after Hu *et al*. 2009) showing the femoral sampling location. (**b**–**j**) Osteohistological features used in assigning ontogenetic stages to each specimen from juvenile to adult demonstrated in femoral thin sections. Note differences in primary vascular density, overall porosity, secondary remodelling and structural organization of the outermost cortex within and between femora representing different degrees of osteohistological maturity. (**b**,**c**) *Eosinopteryx*; (**d**,**e**) *Serikornis*; (**f**,**g**) *Anchiornis*; (**h**) *Aurornis*; (**i**,**j**) *Jeholornis*. Dashed-line rectangles in (**b,d,f,i**) indicate magnified areas in (**c,e,g,j**) respectively. White arrowheads point to lines of arrested growth (lags) present in the external fundamental system (efs). Further abbreviations: avpb, avascular primary bone; c, cortex; es, endosteal surface; flc, fibrolamellar complex; hp, high porosity region; icl, inner circumferential layer; lp, low porosity region; pb, primary bone; po, primary osteon; ps, periosteal surface; pvc, primary vascular canal; sb, secondary bone; so, secondary osteon. Scale bars: 600 µm in (**b,d,f,i**); 300 µm in (**c,e,g,h,j**).

Based on the qualitative assessment of the diaphyseal osteohistology generalized over all sampled elements (Table 1), the ontogenetic stages of the five paravian dinosaur specimens range from juveniles to adults (Fig. 1, Supplementary Figs S1–S3).

Eosinopteryx YFGP-T5197 shows the histocharacteristics of an actively growing juvenile skeleton with high porosity and uniform vascular density in the dominantly primary diaphyseal cortex in most limb bones (Fig. 1b,c). Some elements reveal FLC characteristic of fast growth phases. None of the sampled bones shows LAGs or other growth marks. These features indicate the young age of this specimen, probably less than one year old.

Serikornis PMOL-AB00200 is categorized as a late-juvenile with more advanced osteohistological maturity (Fig. 1d,e) than seen in the bones of *Eosinopteryx* YFGP-T5197. Primary osteons are more mature. While the humerus and femur lack any growth marks, antebrachial bones reveal a single intracortical LAG close to the bone surface, and D1P1 already shows three asymmetrical growth marks. In most sampled bones, decreasing vascularity towards the periosteal surface (Supplementary Fig. S4) indicates decrease in diametric bone growth rate. However, presence of vascular canals in the outermost cortex suggests that considerable diametric growth would have still been possible, had this animal lived longer. The number and spatial distribution of growth marks in some elements, along with the general histological features in the bones lacking growth lines, imply an age not older than two years. Nevertheless, the diverse presence/absence and number of growth marks in different bones highlight the limits of using LAG counts as a proxy for age (see Methods).

Anchiornis YFGP-T5199 represents a sub-adult specimen with histological signs of nearly completed growth. Primary vascularity gradually decreases towards the periosteal surface in most elements (Fig. 1f,g), while in others the outermost cortex is avascular and reminiscent of OCL typically seen in fully grown bird bones^41^. Humerus and antebrachial bones preserve a single intracortical LAG. Although the furcula locally records six closely packed LAGs in an avascular EFS, this element and the entire left wing of YFGP-T5199 are manipulated and the furcula restored from another specimen (U. Lefèvre pers. comm.). Thus, based on the lack of EFS in other elements and the single LAG identified in the longest forelimb bones, this specimen was likely about two years old.

Although *Aurornis* YFGP-T5198 shows poor microstructural preservation, all sampled bones clearly demonstrate its skeletal maturity. Elements reveal a thick avascular outer cortical layer either with avian-like lamellar OCL, or with 1–3 densely spaced LAGs in an EFS (Fig. 1h). Considering that the general number of LAGs is one within the cortex, and 1–3 in the EFS, this adult may have been two to four years old.

Jeholornis YFGP-yb2 shows a peculiar interelement histology pattern with the sampled bones showing sharply contrasting ontogenetic tissue traits. While its hand bones are still actively growing with juvenile-like uniformly high cortical vascularity and no detectable sign of growth rate decrease, the rest of the sampled bones reveal a well-developed EFS with 2–4 LAGs, implying the adulthood of this specimen (Fig. 1i,j). Based on the bones exhibiting EFS, this adult *Jeholornis* is estimated to have been three to five years old.

### Growth rates and precocity ranks of elements

**Figure 2:**
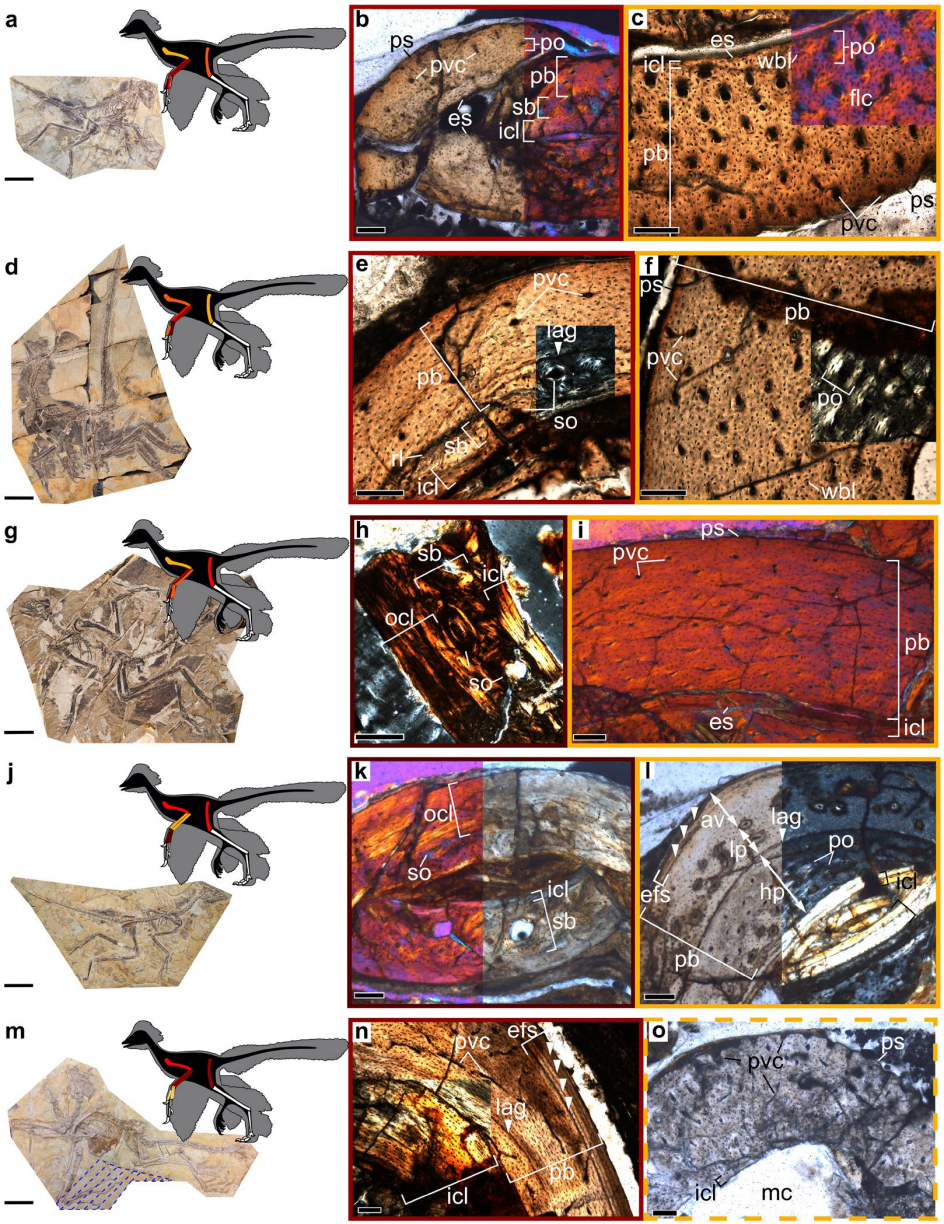
Precocity ranks in the limb bones of paravian dinosaurs assigned on the basis of osteohistological indicators of growth rate and biomechanical demands. (**a**) *Eosinopteryx brevipenna* YFGP – T5197 and thin sections of its (**b**) radius and (**c**) humerus. (**d**) *Serikornis sungei* PMOL-AB00200, and thin sections of its (**e**) ulna and (**f**) femur. (**g**) *Anchiornis huxleyi* YFGP – T5199, and thin sections of its (**h**) first phalanx of digit I (D1P1) and (**i**) humerus. (**j**) *Aurornis xui* YFGP – T5198, and thin sections of its (**k**) D1P1 and (**l**) radius. (**m**) *Jeholornis curvipes* YFGP – yb2 and thin sections of its (**n**) ulna and (**o**) manipulated ‘D1P1’. Colour shading of sampled limb elements lightening up from dark brown to yellow in the schematic paravian silhouettes and in the frames of the histological images represents inferred relative precocity (as defined in the main text) from the most ‘precocial’ (dark brown) to the most ‘altricial’ (yellow) bones within the skeleton. Area marked by blue dashed lines around the left hand bones in *Jeholornis* (**m**) indicates the extent of the reconstructed block. Bones identified as more precocial (**b**,**e**,**h**,**k**) show lower primary vascularity, low to no woven bone content and considerable secondary remodelling referring to slower growth in their primary tissue and higher mechanical demands, respectively. By contrast, more altricially growing elements (**c**,**f**,**i**,**l**,**o**) show higher primary porosity, woven bone, and low or no secondary remodelling. Note the controversial histological pattern between the mature, large ulna (**n**) and the still actively growing small ‘D1P1’ (**o**) in *Jeholornis*. Abbreviations: av, avascular region; mc, medullary cavity; ocl, outer circumferential layer; rl, resorption line; wbl, woven bone lacunae. Further abbreviations as in Fig. 1. Scale bars: 5 cm in skeletal specimens, 100 µm in thin sections.

Representing different ontogenetic stages, these paravian dinosaurs provide snapshots of intraskeletal growth dynamics in different phases of their respective growth trajectory. The distribution of precocity ranks among different bones within their skeleton is also diverse (Fig. 2a,d,g,j,m).

Even though in the juvenile skeleton of *Eosinopteryx* the antebrachial bones are the second longest sampled elements (Supplementary Table S1), they show comparatively slow primary cortical growth with low woven bone content and scarce vascularity, a relatively thick ICL, and extensive secondary remodelling in the inner cortical half. In fact, radius histology suggests that this element had the lowest growth rate among all sampled bones (Fig. 2b), while growth dynamics of the ulna is more comparable to that of the relatively slow growing but much smaller metacarpal II. By contrast, the similarly sized humerus with the highest porosity, woven bone content, and locally FLC in the primary cortex, abundant canals opening onto the undulating periosteal surface, and no remodelling, is evidently the fastest, most dynamically growing element (Fig. 2c). The longest sampled bone, the femur, also reveals rapid and continuous growth regionally with FLC, but a more mature histology with well-developed primary osteons (Fig. 1c), relatively thicker ICL, and remnants of secondarily remodelled inner cortical region. The high woven bone content and primary porosity combined with the remodelled inner cortex in the shortest sampled bone, D1P1, imply fast growth but extensive functional deployment of this element. This histological pattern suggests that the humerus was the most altricially developing bone in the limbs.

In the late-juvenile or subadult skeleton of *Serikornis* the longest sampled element, the femur (Supplementary Table S1) had apparently the highest growth rate (Fig. 2f) followed by the second-longest humerus. They are both characterized by a thin ICL and notable slow-down of growth with decreasing vascularity from about 2/3 of the cortex but without growth marks or secondary remodelling (Fig. 2c). However, similarly to *Eosinopteryx*, the ulna shows extensive remodelling in the inner cortex, sparse primary vascularity, and a single LAG running close to the remodelled area (Fig. 2e). Although it is poorly preserved, regionally extensive remodelling up close to the periosteal surface also occurs in the radius. D1P1 reveals two incomplete LAGs partially removed by cortical drift during asymmetrical diametric bone growth^72^, and a less distinct growth mark in between these LAGs. However, its woven bone content and vascularity in other cortical regions imply that the short D1P1 grew faster than the much longer antebrachial bones in the recorded ontogenetic period. Interestingly, metacarpals which share the same anatomical region with, and are longer than D1P1, show sparse primary vascularity and extensive remodelling indicating slower growth and possibly higher mechanical deployment than in D1P1. Thus, a relatively precocial development is suggested for the antebrachial and metacarpal elements as compared to the rest of the sampled bones.

In the sub-adult *Anchiornis* the humerus and the femur are the longest sampled bones (Supplementary Table S1), but their histology is disparate. The humerus shows a largely uniform microstructure with exclusively primary cortex and moderate vascular density that slightly and gradually decreases periosteally (Fig. 2i). By contrast, the femur shows extensive asymmetries along its preserved circumference in the extent of secondary remodelling and vascular densities with regionally avascular outer cortex (Fig. 1f,g). These asymmetries indicate considerable cortical drift likely associated with the characteristic slight anterior curvature in the femoral shaft, as opposed to the straight mid-diaphysis of the humerus. Nevertheless, the single but incomplete LAG close to the ICL also refers to some cortical drift in the humerus. Woven bone content suggests that the femur grew faster than the humerus up to its current size with a sudden drop in growth speed on one side and a more gradual decrease on the opposing cortical side. Ulnar histology is similar to that of the humerus, while the radius shows a slower growth, more abrupt decrease in vascularity in the outer cortex, and secondary remodelling close to the medullary cavity. D1P1 shows sparse primary vascularity and extensive remodelling (Fig. 2h) contrasting the almost exclusively primary cortex of the metacarpal bones. However, differences in the extent of remodelling among these bones likely originate from the more distal and more proximal sampling location in relation to the arithmetic mid-diaphysis in the radius and D1P1, respectively (Supplementary Table S2). No LAGs or other growth marks can be observed in the sampled antebrachial and hand bones.

In the fully-grown *Aurornis*, the humerus and femur show extremely similar histology with regionally different primary vascular densities and asymmetrically remodelled inner cortex reaching up to a mid-cortical incomplete LAG. Thus, similar growth rates and functional demands are inferred in these elements. The slightly shorter radius and ulna (Supplementary Table S1) both have a cortex thickness regionally varying by a factor of 1.7, relatively low woven bone content, locally varying vascular densities, a double-LAG running in the mid-cortex, and almost no remodelling. The thickest cortical region in the radius preserves an inner part up to the LAG with notably high vascular density, that represents remnants of a faster growing juvenile tissue (Fig. 2l). Such asymmetries are unexpected given the comparatively straight shaft of antebrachial bones and the mid-diaphyseal sampling location. All hand bones have entirely remodelled inner cortex, while the primary outer half is composed of an inner sparsely vascularized and (or exclusively) an outer avascular OCL-like layer (Fig. 2k). These features suggest similar growth rates in similarly sized elements but potentially lower biomechanical demands in the antebrachial bones in the recorded ontogenetic period.

Besides the controversial hand bones in *Jeholornis*, the humerus, which is the most robust and longest element in the skeleton (Supplementary Table S1), grew the fastest, based on its highest vascular density and woven bone content. Otherwise, the humerus, antebrachial bones, and the considerably shorter femur have quite uniform adult histology. The extremely thick ICL makes up 1/3 to regionally more than ½ of the total cortex thickness and is perforated by mostly radial vascular canals which regionally invade the primary cortex too. Remodelling is generally low. The ulna (Fig. 2n) and the femur have the highest proportion of circumferentially-oriented primary vascular canals among all bones investigated in this study, which are dominated by longitudinal canals. All four elements reveal one intracortical LAG and an EFS with 2–4 LAGs making up about 10% of total cortex thickness. Vasculature profiles (Supplementary Fig. S4) suggest that growth rate dropped suddenly before the deposition of the first LAG in the EFS. By strong contrast, ‘D1P1’ is in a growth burst with a thin ICL and a rugose periosteal surface perforated by several wide-open vascular canals (Fig. 2o). It lacks growth marks but reveals secondary remodelling in the inner cortex, although the extent of remodelling is difficult to discern. ‘Metacarpals II & III’ have more advanced histological maturity with well-developed ICL, growth marks, and remodelling to different degrees but no EFS. Strangely, these small hand bones show higher woven bone content (locally FLC) and vascular densities than the largest bone, the humerus, implying that they grew faster in the recorded ontogenetic period. This ongoing hand growth sharply contrasting the finalized growth and likely advanced age recorded in the longer limb bones suggests unrealistic heterochronic processes with an extreme allometric growth pattern or a pathologic condition. However, this pattern is much more likely to provide the independent osteohistological evidence for the original suspicion of Lefèvre *et al*.^18^ that, based on intensity differences in X-ray images, the left hand of YFGP-yb2 has been reconstructed with bones taken from another animal (see also Discussion).

### Quantified histovariability

The unlikely growth dynamics and suspected forgery in the hand bones of *Jeholornis* required the combined interpretation of two different sets of quantitative analyses: 1) with the inclusion of all five specimens but with the exclusion of hand bones; and 2) with the inclusion of hand bones but with the exclusion of *Jeholornis* (see details in Supplemetary Information).

**Figure 3:**
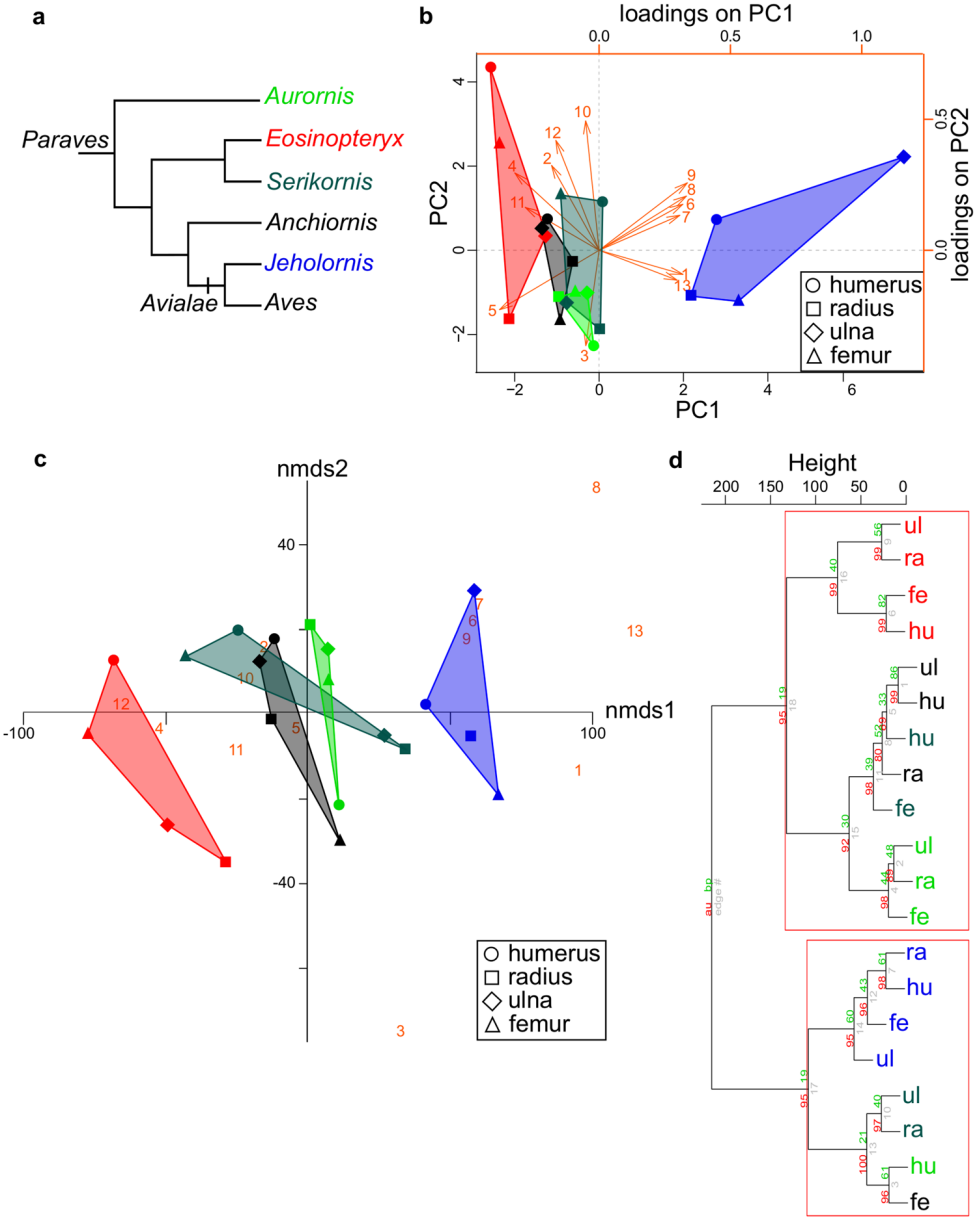
Simplified phylogeny of the studied taxa and the visual output of different multivariate analyses of histological characters with the exclusion of hand bones. (**a**) Phylogenetic interrelationships among the sampled taxa and modern birds (Aves) (based on Lefèvre *et al*. 2017). (**b**) PC1-PC2 scatterplot with indication of variable loadings (arrows and numbers in orange). (**c**) Two-dimensional NMDS scatterplot with indication of the relative effect of variables on the ordination of elements (numbers in orange). Shaded polygons indicate distribution of coherent elements by specimen. (**d**) Cluster dendrogram based on Euclidean distances applying Ward’s method with significance values (%) indicated on each cluster branch. Red rectangles mark significant clusters. Note that only elements of the adult avialan *Jeholornis* (blue) appear as a distinct group with every method, while the coherence of elements of the juvenile *Eosinopteryx* remains unrecognized by PCA. Colour codes of specimens as in a. Number codes of variables as given in Table 3. Further abbreviations: fe, femur; hu, humerus; ra, radius; ul, ulna. See also Figure S5 for the results of multivariate analyses with hand bones but with the exclusion of *Jeholornis*.

PCA, NMDS and cluster analyses of the measured histological characters (Table 3, Supplementary Table S2) in all five specimens with the exclusion of hand bones uniformly emphasize that *Jeholornis* is histologically distinct from all other taxa. Besides *Jeholornis*, only elements of *Eosinopteryx* display some degree of specimen-level coherence, although this separation is less distinct and less consistent than those of *Jeholornis* among different multivariate visualizations (Fig. 3). However, PERMANOVA detected significant distinction only between the juvenile (*Eosinopteryx*) and adult (*Aurornis* and *Jeholornis*) ontogenetic categories, whereas it does not support taxonomic or homologous element groups in the multivariate dataset. As both PC1 and NMDS1 scores are largely influenced by vascularity features and ICL-EFS thickness %, and they correlate well with measures of size across individuals, they seem to reflect allometric changes in these histological features related to size increase. This may explain the clear distinction of *Jeholornis*, the largest sampled specimen. By contrast, PC2 and NMDS2 correlate with the qualitatively assigned precocity ranks as well as with sampling location, the latter two of which also strongly and negatively correlate with each other. However, PCA eigenvalues and variation partitioning of PCA and NMDS scores indicate a low explanatory power of these considered factors in total histological variability (~50%). In the other specimens, PCA, NMDS and cluster analyses all imply higher intraskeletal than interspecimen histodiversity. However, no homologous element-specific distribution of histological traits can be detected, and elements do not show consistent intraskeletal precocity ranks, either.

Analyses with hand bones but without *Jeholornis* also reveals a great intraskeletal histodiversity with even less element-specific distribution pattern (Supplementary Fig. S5). Nevertheless, elements of *Eosinopteryx* are separated in all multivariate visualizations, and their specimen-level coherence is also supported by PERMANOVA with both ontogenetic stage and taxonomic grouping factors. No other specimen shows any distinction from the rest with the applied methods. In contrast to the predominant importance of size in the analyses without hand bones, correlation analyses on PCA and NMDS scores suggest that osteohistological variability reflects growth dynamics of short vs. long bones, revealing a positive relationship between precocity ranks and element size. Despite that, homologous elements do not form distinct groups in these analyses, either. Although precocity ranks are reflected in the internal structuring of the cluster dendrogram, and their importance is highlighted by variation partitioning as well, the latter also reveals that these ranks explain only a very low proportion of the detected variance in bone microstructure (~25–50%). Thus, inclusion of hand bones in these analyses further reduced the efficiency of the considered explanatory factors in accounting for the histovariability pattern.

## Discussion

Although these dinosaurs show the general ‘dinobird’ bauplan with similar bone length proportions within the elongate forelimbs and hind limbs (Supplementary Table S1), the sampled bones reveal diverse growth dynamics that do not adhere to an element-specific consistent pattern across specimens. Their differing ontogenetic stages clearly contribute to this variability, however, subadult and adult specimens with nearly or entirely completed growth also reveal variable intraskeletal growth dynamics. Accounting for differences in element lengths and cortical robustness, growth trajectories of individual bones refer to a variety of intraskeletal allometric and isometric growth patterns that likely reflect interspecific diversity in growth strategies.

Inferred growth rate differences and relative precocity degrees in the limb bones of *Eosinopteryx* reveals pronounced allometries, in which antebrachial bones were most likely disproportionately long already at much earlier ontogenetic stages. Thus, the radius and ulna either had an early growth burst not recorded in the preserved cortex or they grew slowly but were already longer than the humerus and maybe even the femur at hatching. Either of these allometric developmental patterns suggests a precocial distal forelimb and more altricial proximal limb bones in *Eosinopteryx*. However, as the humerus would have clearly outgrown the antebrachial bones had this juvenile specimen lived longer, a considerable shift in forelimb function likely occurred during the ontogeny of *Eosinopteryx*. It is unknown whether the apparently higher growth rate was maintained long enough in the humerus to eventually outgrow the femur potentially implying improved aerial capacities, or the femur remained longer indicating prevalence of bipedalism. Nevertheless, the forelimb/hind limb length ratio (as defined in Supplementary Table S1) in this juvenile *Eosinopteryx* is already equal to that of the subadult YFGP-T5199 specimen of *Anchiornis*, a presumably volant taxon^73,74^. Furthermore, as opposed to its original morphology-based subadult-adult ontogenetic assignment^16^, the juvenile nature of *Eosinopteryx* YFGP-T5197 and its osteohistology-based allometric growth patterns caution about its possible taxonomic interpretations.

Histodiversity in *Serikornis* refers to a largely isometric growth of the longer limb bones with a positive relationship between element size and inferred growth rates. However, the unexpectedly altricial development of D1P1, the shortest sampled bone in the skeleton, implies a distinct role of this element in the hand. The robust claw on D1P1 can provide an additional clue to functional interpretations, such as scansorial and/or raptorial grasping adaptations^40^, the efficiency of which might have improved as ontogeny progressed. Considering the late juvenile to subadult ontogenetic stage of this specimen, proportions could have slightly shifted towards a more robust humerus and femur relatively to the antebrachium later during ontogeny. Whether this proportional change would have been pronounced enough to indicate a functional shift in the forelimbs remains unknown. However, length difference between the humerus and the femur would likely have remained constant or even increased later during development, emphasizing a potentially low aerial capability, consistent with its plumage attributes^42^, and predominance of bipedal locomotion in this taxon.

Limb bone histology in the sub-adult *Anchiornis* YFGP-T5199 suggests a moderately allometric intraskeletal growth pattern. Extensive remodelling indicates a precocial femoral development, albeit a fast growth period is still revealed in the primary tissue. This implies that the femur reached its subadult dimensions growing faster than most other elements but was likely extensively loaded already earlier in ontogeny. The femur of the holotype IVPP V14378^4^, the single osteohistological slide ever reported in *Anchiornis* besides the current study, supports this deduction. The holotype is considerably smaller than YFGP-T5199, and its femoral histology also refers to its earlier, possibly late juvenile, ontogenetic stage. In IVPP V14378, the femur is longer than the humerus, albeit already with similar extent of remodelling near the ICL^4^ as detected in the femur of YFGP-T5199. This further demonstrates that the initial fast growth of the femur ensured its early functional maturation and suggests that bipedal locomotion was already prevalent in early juvenile stages. In YFGP-T5199, a subsequent decrease in the femoral growth rate enabled the humerus, growing at lower but more constant rate, to eventually catch up with the femur. The negligible remodelling in the long forelimb bones as opposed to the extensive remodelling in the femur indicates lower musculoskeletal strains on the forelimbs at this ontogenetic stage. Accounting for sampling location as another possible factor responsible for remodelling differences, growth dynamics of the antebrachial and hand elements seems to conform to their length ratios. Little or no allometric size change among the sampled elements is expected during later ontogeny.

Nevertheless, as evidenced by multiple specimens reported so far^4,75,76^, a considerable proportional variation exists among the limb elements in *Anchiornis*. For instance, the humerus/femur ratio varies between 0.8 and 1.05, and does not seem to show any trend with respect to specimen size. This proportional variability in *Anchiornis* either reflects real intraspecific variation in allometric growth patterns or preservational and consequently measurement artefacts^76^. Multielement osteohistological analyses of multiple specimens could test the biological origin of this proportional variation. As forelimb/hind limb ratio indicates aerial skills^73–75^, osteohistological studies would also be important for inferring the aerodynamic performance-range of *Anchiornis*^73–75,77^.

Histodiversity in *Aurornis* implies a largely isometric growth pattern with the longest sampled bones, the humerus and femur, preserving a precocial growth record in the adult stage, albeit with fast initial growth. Vascular features suggest similar growth rates in the antebrachial bones, femur and humerus up to the first LAG. Thereafter, growth rate markedly decreased and growth continued slower in the radius and ulna, while the femur and humerus apparently retained a higher growth rate longer, with a more abrupt decrease when final size was reached. Thus, these proximal limb bones likely outgrew antebrachial elements to acquire their functional maturity already early in ontogeny. The considerable but slow bone accumulation after the initial fast growth phase, and the negligible remodelling in the radius and ulna imply less loading effect on the growth of these elements. However, detected difference in remodelling features may be related to the more distal sampling location in the femur and humerus (Supplementary Table S2). As YFGP-T5198 represents a fully-grown *Aurornis*, its limb proportions enable conclusive studies on the potential aerodynamic performance of this likely volant animal^73,74^.

The fake origin of the left hand bones in the avialan *Jeholornis* YFGP-yb2^18^ is also evidenced by its conflicting intraskeletal histology, which suggests an allometric growth pattern that is not supported by the limb proportions in other *Jeholornis* specimens. Wing element length ratios in the fully-grown *Jeholornis* YFGP-yb2 corresponds well with those described in other *Jeholornis* specimens: *Jeholornis prima* IVPP V 13274 (holotype^15^), IVPP V 13353^78^, LPM0193 (originally described as *Shenzhouraptor sinensis* by Ji *et al*.^79^), and *Jeholornis* sp. STM 2–51^80^. Among these, LPM0193 is the smallest and has been identified as a juvenile^81–83^. If the left hand bones and their inferred growth pattern in YFGP-yb2 were real, hand bones in the juvenile LPM0193 would be relatively shorter, starting to grow fast only after the long wing bones are fully grown. This, however, is not the case. In fact, general histological and cross-sectional features of ‘D1P1’ in YFGP-yb2 imply that this bone is not even a phalanx but rather a more proximal limb bone of a fast growing juvenile animal of unknown taxonomic status. The preserved primary cortex in the other sampled bones of YFGP-yb2 suggests the highest growth rate in the most robust humerus, and similarly lower rates in the antebrachial bones and the femur. This implies that the considerable size difference between the shorter femur and the longer radius and ulna already existed earlier in ontogeny and hence similar growth rates maintained these proportions throughout development. The humerus, having similar length as, but being more robust than the radius and ulna in the adult YFGP-yb2, likely grew isometrically with the antebrachial elements by its higher diametric growth rate.

Disregarding its fake left hand bones, limb elements of *Jeholornis* YFGP-yb2 reveal a distinct osteohistology that readily distinguishes them from all other specimens studied here but that largely, though not entirely, corresponds with previous reports in *Jeholornis prima* IVPP 13353^3^ and *Jeholornis* sp. STM 2–51^9^. For instance, the proportion of transverse to longitudinal primary vascular canals in YFGP-yb2, especially in its ulna and femur, is higher than in any element studied in the non-avialan specimens. As opposed to a pure longitudinal vascularization reported in the femur of *Jeholornis prima* IVPP 13353^3^, reticular vasculature has also been detected both in the ulna and femur of *Jeholornis* sp. STM 2–51^9^. Our quantitative results support the hypothesis that these vascular features are associated with size and hence represent allometric changes in the vascular architecture^84^. Whether a more complex vascular pattern compared to a predominantly longitudinal architecture involves differences in bone growth rate is not clear^85–87^. However, vascular densities and relative woven bone content in *Jeholornis* elements do not imply higher growth rates than in the homologous bones of the subadult *Anchiornis* or adult *Aurornis*, both of which apparently attain smaller adult body size than *Jeholornis*.

The most conspicuous and distinctive histological feature in *Jeholornis* YFGP-yb2 is the extremely thick ICL in all sampled elements, especially in the femur. Although ulnar histology of *Jeholornis* sp. STM 2–51^9^ shows similarly thickened ICL, the femur of the same specimen does not, which may support a pathologic origin of the condition in *Jeholornis* YFGP-yb2. However, there is neither macroscopic deviation visible in the morphology of these bones, nor other typical histological signs of bone pathologies, such as lesions, periosteal bone thickening, extreme remodelling or osteoporotic cortex^88–91^. ICL as thick as in the wing bones of *Jeholornis* YFGP-yb2 has also been demonstrated in the humerus, radius, and ulna of *Confuciusornis*, yet again not in the femur^3,92,93^. Robust ICL characterizes the femur and tibiotarsus of two diving birds, a Cretaceous loon TTU P 9265^94^ and the extant common murre (*Uria aalge*)^95^, respectively. As the possible cause of this notably thick ICL has not been discussed in these taxa, it remains unclear whether the extremely thick femoral ICL is pathological or a characteristic feature of the species *Jeholornis curvipes*. However, a medullary bone origin for this structure is very unlikely, as it lacks the characteristic high porosity and woven bone content of medullary bone^96,97^.

Because *Jeholornis* is the only avialan included in the present study, it is possible that these distinctive histological features are associated with powered flight, contrasting the comparatively poor aerial capabilities of the more basal paravians *Eosinopteryx*, *Aurornis*, *Anchiornis* and *Serikornis*^42^. For instance, the higher proportion of transverse vascular canals in *Jeholornis* limb bones could be a functional adaptation to resist powered flight-induced torsional loads, as suggested for the laminar vascular architecture frequently seen in modern bird wing bones^95,98,99^. However, re-investigation of this relationship on a broader spectrum of flying taxa, including bats, found no statistically supported correlation between bone laminarity and flight^87^. Thus, histological correlates of aerial locomotion are not yet well-understood, and need further study in extant volant vertebrates to draw firm conclusions on their adaptive and phylogenetic significance in dinosaur-bird transitional forms. As strong functional association between powered flight and bone regulation, remodelling, metabolism and fusion was also revealed at a genetic level^100^, this endeavour promises new insights into the origin and evolution of avian powered flight, complementing gross-morphological studies^101–104^.

Our quantitative results are largely but not entirely in line with the qualitative observations. Whereas the unique histology of the avialan *Jeholornis* consistently appeared in the exploratory analyses, numerical tests did not confirm the statistical validity of taxon-specific histology in any of the taxa, when all five specimens were included. The exclusion of *Jeholornis* and inclusion of hand bones in the analyses resulted in a taxon-specific distinction of *Eosinopteryx* which, however, is most likely due to its juvenile histology. Interestingly, homologous elements do not show a uniform histology, whereas element size and related allometric traits are more consistent with the quantified histodiversity patterns in these paravians. Sampling location also has a considerable effect. Although largely based on the same osteohistological characters as measured for the analyses, the qualitatively assessed ontogenetic stages and precocity ranks only partially accounted for the quantified histodiversity. The >50% consistently unexplained histovariability highlights the importance of unexplored and/or more complex network of factors, such as locomotion-related adaptations, in shaping the investigated osteohistological characters. Still, despite the structural and functional complexity of bone tissues, our results imply that qualitative assessments of ontogeny and intraskeletal growth allometries create an important conceptual framework for investigating the relative contribution of these interrelated factors.

If captured in the right ontogenetic window, osteohistology-based intraskeletal growth dynamics may be informative of actual precocial – altricial limb usage. In a fluorescent-labelling study of periosteal bone growth rates in different fore- and hind limb elements of the mallard (*Anas platyrhynchos*), Castanet *et al*.^24^ showed that, consistent with the negative relationship between growth and functional maturity^25,69,70^, the precocially developing leg bones grow considerably slower than the altricially developing humerus, and that these bones accordingly reveal a disparate histology. In addition, secondary remodelling appeared from as early on as 7 weeks posthatching in the precocial hind limb elements, whereas the altricial humerus did not reveal any remodelling up to the time of sacrifice (25 weeks). This finding supports our precocity rank assumption that the extent of remodelling is also associated with the extent of biomechanical demands through musculoskeletal performance. A negative relationship between longitudinal bone growth rate and precocity degree has also been demonstrated in the comparative osteohistological study of the femur of the highly precocial ring-necked pheasant chicks (*Phasianus colchicus*) performing physically demanding terrestrial locomotion, and the semi-aquatic mallard chicks having less mechanical constraints on their femur^25^. Suppression of longitudinal bone growth by dynamic loading in juvenile animals has also been shown experimentally^105,106^. However, as mechanical load can also induce osteogenesis and facilitate periosteal bone deposition and mineralization even in juveniles^105,107–111^, this negative relationship may be obscured. Although both, periosteal bone growth and secondary remodelling, can be induced by exercise, a trade-off between them was observed in the limb bones of juvenile sheep: higher induced periosteal growth was coupled with lower remodelling and vice versa in the proximal and distal midshaft, respectively^110^. Inconsistent results on the relationship between diametric and longitudinal bone growth and increased mechanical loading (positive in juvenile rats^112^ vs. negative in juvenile rats^105^ and chickens^106^) further complicate the possible interpretations of intraskeletal growth dynamics in these bird-like dinosaurs. Even within the physiological loading regimes, this relationship may change depending on the static vs. dynamic nature, magnitude, frequency and duration of load in the context of hormonal, nutritive and ontogenetic status^113^. Finally, while epiphyseal histology related to longitudinal bone growth has been investigated to explore how these important functional aspects are mirrored in the avian precocial – altricial developmental spectrum^20,25,46,114^, their potential effects on the mid-diaphyseal bone tissue and diametric growth have yet to be studied. Thus, our results on the osteohistological variability in these paravian taxa cannot yet be put in the context of effective ontogenetic locomotor strategies.

In summary, intraskeletal histovariability suggests diverse growth patterns in the limb bones of these paravian dinosaurs. As avian ontogenetic patterns are strongly related to flight^115^, our study also emphasizes the importance of unraveling ontogenetic stages and postnatal intraskeletal growth dynamics of bird-like dinosaurs in the studies of aerodynamic performance and evolution of avian flight. Our ongoing research on extant birds will explore osteohistological correlates of ontogenetic locomotor strategies; a crucial basis to investigate these functional relationships in paravian dinosaurs.

## Electronic supplementary material

Supplementary Information

Supplementary Table S2
