# Publisher Correction: Intraskeletal histovariability, allometric growth patterns, and their functional implications in bird-like dinosaurs

**DOI:** 10.1038/s41598-018-20640-6

**Published:** 2018-03-06

**Authors:** Edina Prondvai, Pascal Godefroit, Dominique Adriaens, Dong-Yu Hu

**Affiliations:** 1 Evolutionary Morphology of Vertebrates, Department of Biology, Ghent University, Ghent, Belgium; 2 Royal Belgian Institute of Natural Sciences, Directorate ‘Earth and History of Life’, Brussels, Belgium; 3 Paleontological Institute, Shenyang Normal University, Key Laboratory for Evolution of Past Life in Northeast Asia, Ministry of Land and Resources, Shenyang, China

Correction to: *Scientific Reports* 10.1038/s41598-017-18218-9, published online 10 January 2018

The original PDF version of this Article contained an error in the order of the Figures and Figure Legends. Figures 1, 2 and 3 were published as Figures 3, 1 and 2 respectively. This has now been corrected in the PDF; the HTML version of the paper was correct from the time of publication.

This Article also contained errors in the Results section under subheading ‘Ontogenetic stages and estimated ages’.

“**Eosinopteryx**

YFGP-T5197 shows the histocharacteristics of an actively growing juvenile skeleton with high porosity and uniform vascular density in the dominantly primary diaphyseal cortex in most limb bones (Fig. 1b,c). Some elements reveal FLC characteristic of fast growth phases. None of the sampled bones shows LAGs or other growth marks. These features indicate the young age of this specimen, probably less than one year old.

**Serikornis**

PMOL-AB00200 is categorized as a late-juvenile with more advanced osteohistological maturity (Fig. 1d,e) than seen in the bones of Eosinopteryx YFGP-T5197. Primary osteons are more mature. While the humerus and femur lack any growth marks, antebrachial bones reveal a single intracortical LAG close to the bone surface, and D1P1 already shows three asymmetrical growth marks. In most sampled bones, decreasing vascularity towards the periosteal surface (Supplementary Fig. S4) indicates decrease in diametric bone growth rate. However, presence of vascular canals in the outermost cortex suggests that considerable diametric growth would have still been possible, had this animal lived longer. The number and spatial distribution of growth marks in some elements, along with the general histological features in the bones lacking growth lines, imply an age not older than two years. Nevertheless, the diverse presence/absence and number of growth marks in different bones highlight the limits of using LAG counts as a proxy for age (see Methods).

**Anchiornis**

YFGP-T5199 represents a sub-adult specimen with histological signs of nearly completed growth. Primary vascularity gradually decreases towards the periosteal surface in most elements (Fig. 1f,g), while in others the outermost cortex is avascular and reminiscent of OCL typically seen in fully grown bird bones41. Humerus and antebrachial bones preserve a single intracortical LAG. Although the furcula locally records six closely packed LAGs in an avascular EFS, this element and the entire left wing of YFGP-T5199 are manipulated and the furcula restored from another specimen (U. Lefèvre pers. comm.). Thus, based on the lack of EFS in other elements and the single LAG identified in the longest forelimb bones, this specimen was likely about two years old.

Although Aurornis YFGP-T5198 shows poor microstructural preservation, all sampled bones clearly demonstrate its skeletal maturity. Elements reveal a thick avascular outer cortical layer either with avian-like lamellar OCL, or with 1–3 densely spaced LAGs in an EFS (Fig. 1h). Considering that the general number of LAGs is one within the cortex, and 1–3 in the EFS, this adult may have been two to four years old.

**Jeholornis**

YFGP-yb2 shows a peculiar interelement histology pattern with the sampled bones showing sharply contrasting ontogenetic tissue traits. While its hand bones are still actively growing with juvenile-like uniformly high cortical vascularity and no detectable sign of growth rate decrease, the rest of the sampled bones reveal a well-developed EFS with 2–4 LAGs, implying the adulthood of this specimen (Fig. 1i,j). Based on the bones exhibiting EFS, this adult Jeholornis is estimated to have been three to five years old.”

now reads:

“Eosinopteryx YFGP-T5197 shows the histocharacteristics of an actively growing juvenile skeleton with high porosity and uniform vascular density in the dominantly primary diaphyseal cortex in most limb bones (Fig. 1b,c). Some elements reveal FLC characteristic of fast growth phases. None of the sampled bones shows LAGs or other growth marks. These features indicate the young age of this specimen, probably less than one year old.

Serikornis PMOL-AB00200 is categorized as a late-juvenile with more advanced osteohistological maturity (Fig. 1d,e) than seen in the bones of Eosinopteryx YFGP-T5197. Primary osteons are more mature. While the humerus and femur lack any growth marks, antebrachial bones reveal a single intracortical LAG close to the bone surface, and D1P1 already shows three asymmetrical growth marks. In most sampled bones, decreasing vascularity towards the periosteal surface (Supplementary Fig. S4) indicates decrease in diametric bone growth rate. However, presence of vascular canals in the outermost cortex suggests that considerable diametric growth would have still been possible, had this animal lived longer. The number and spatial distribution of growth marks in some elements, along with the general histological features in the bones lacking growth lines, imply an age not older than two years. Nevertheless, the diverse presence/absence and number of growth marks in different bones highlight the limits of using LAG counts as a proxy for age (see Methods).

Anchiornis YFGP-T5199 represents a sub-adult specimen with histological signs of nearly completed growth. Primary vascularity gradually decreases towards the periosteal surface in most elements (Fig. 1f,g), while in others the outermost cortex is avascular and reminiscent of OCL typically seen in fully grown bird bones41. Humerus and antebrachial bones preserve a single intracortical LAG. Although the furcula locally records six closely packed LAGs in an avascular EFS, this element and the entire left wing of YFGP-T5199 are manipulated and the furcula restored from another specimen (U. Lefèvre pers. comm.). Thus, based on the lack of EFS in other elements and the single LAG identified in the longest forelimb bones, this specimen was likely about two years old.

Although Aurornis YFGP-T5198 shows poor microstructural preservation, all sampled bones clearly demonstrate its skeletal maturity. Elements reveal a thick avascular outer cortical layer either with avian-like lamellar OCL, or with 1–3 densely spaced LAGs in an EFS (Fig. 1h). Considering that the general number of LAGs is one within the cortex, and 1–3 in the EFS, this adult may have been two to four years old.

Jeholornis YFGP-yb2 shows a peculiar interelement histology pattern with the sampled bones showing sharply contrasting ontogenetic tissue traits. While its hand bones are still actively growing with juvenile-like uniformly high cortical vascularity and no detectable sign of growth rate decrease, the rest of the sampled bones reveal a well-developed EFS with 2–4 LAGs, implying the adulthood of this specimen (Fig. 1i,j). Based on the bones exhibiting EFS, this adult Jeholornis is estimated to have been three to five years old.”

This has now been corrected in the PDF and HTML versions of the Article.

