# Supplementary material for: Intraskeletal histovariability, allometric growth patterns, and their functional implications in bird-like dinosaurs
Source: Sci Rep. 2018 Jan 10;8:258. doi: 10.1038/s41598-017-18218-9 (PMC5762864; doi:10.1038/s41598-017-18218-9)
Supplement: Supplementary file 1 — Supplementary Information [file 41598_2017_18218_MOESM1_ESM.pdf]

SUPPLEMENTARY INFORMATION

‘**Intraskeletal histovariability, allometric growth patterns, and their functional implications in bird-like dinosaurs’**

by

*Edina Prondvai, Pascal Godefroit, Dominique Adriaens, and Dong-Yu Hu

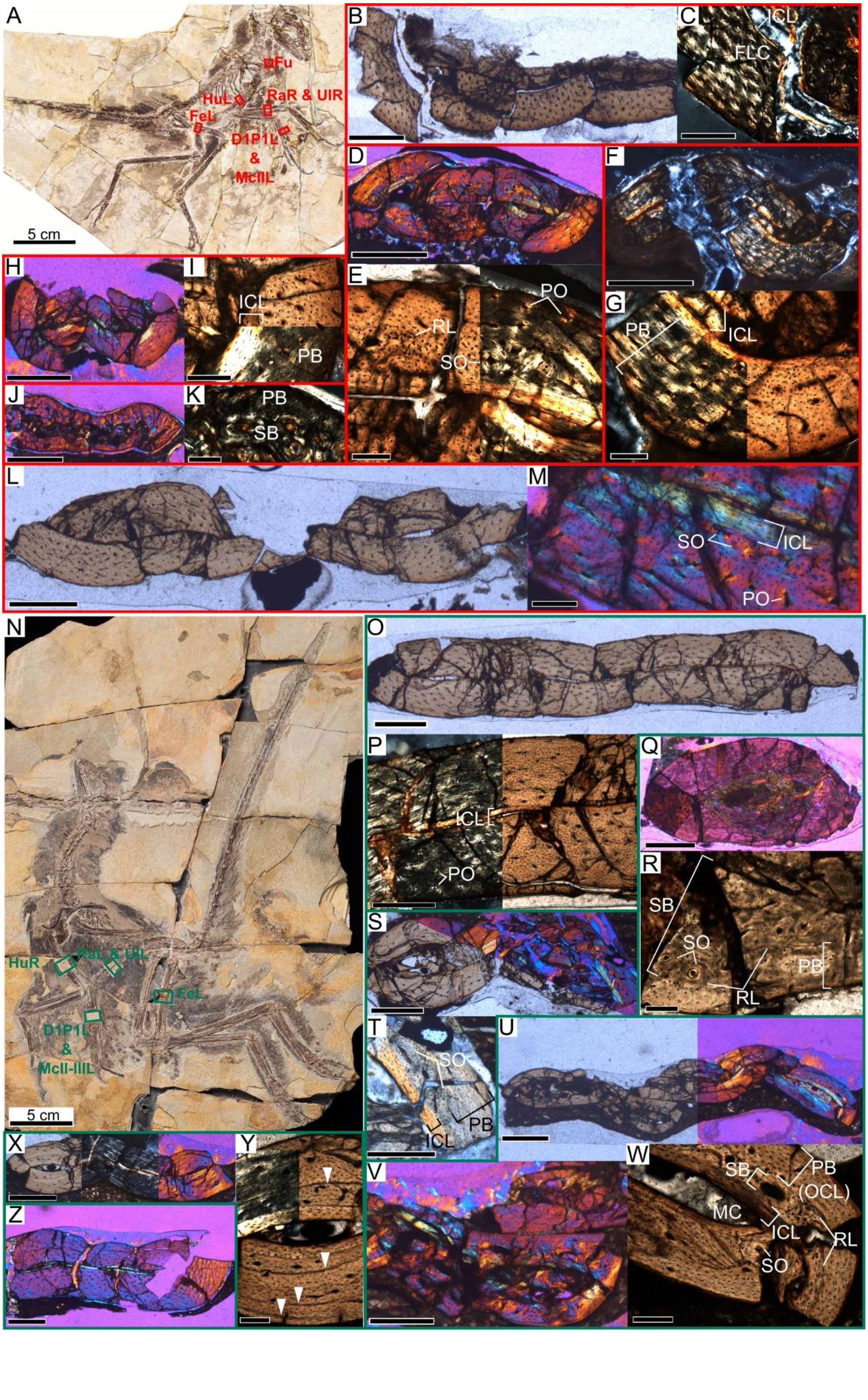

A
Fu
HuL
RaR & UIR
FeL
D1P1L
&
McIIL
5 cm
B
C
ICL
FLC
D
F
H
I
ICL
PB
E
PO
RL
SO
G
PB
ICL
J
K
PB
SB
L
M
SO
ICL
PO
N
O
P
ICL
PO
Q
R
SB
SO
PB
RL
S
HuR
RaL & UlL
FeL
D1P1L
&
McII-IIIL
5 cm
T
SO
PB
ICL
U
X
Y
Z
V
W
SB
PB
(OCL)
MC
ICL
RL
SO

Figure S1. Intraskeletal histodiversity in the juvenile *Eosinopteryx brevipenna* YFGP-T5197 and the late-juvenile – subadult *Serikornis sungei* PMOL-AB00200. A, *Eosinopteryx* YFGP-T5197 skeleton with indication of sampling locations and histological thin sections of its B-C, humerus; D-E, radius; F-G, ulna; H-I, McII; J-K, D1P1; and L-M, femur. N, *Serikornis* PMOL-AB00200 skeleton with indication of sampling locations and histological thin sections of its O-P, humerus; Q-R, radius; S-T, ulna; U-W, metacarpal II-III; X-Y, D1P1; and Z, femur. White arrowheads point to lines of arrested growth (LAGs). Abbreviations as in Figures 1-2 and Figure S1. Colour codes of specimens as in Figure 3. Scale bars: 600 μm in B,D,F,H,J,L,O,Q,S,U,X,Z; 300 μm in C,P,T,V; 100 μm in E,G,I,K,M,R,Y,W.

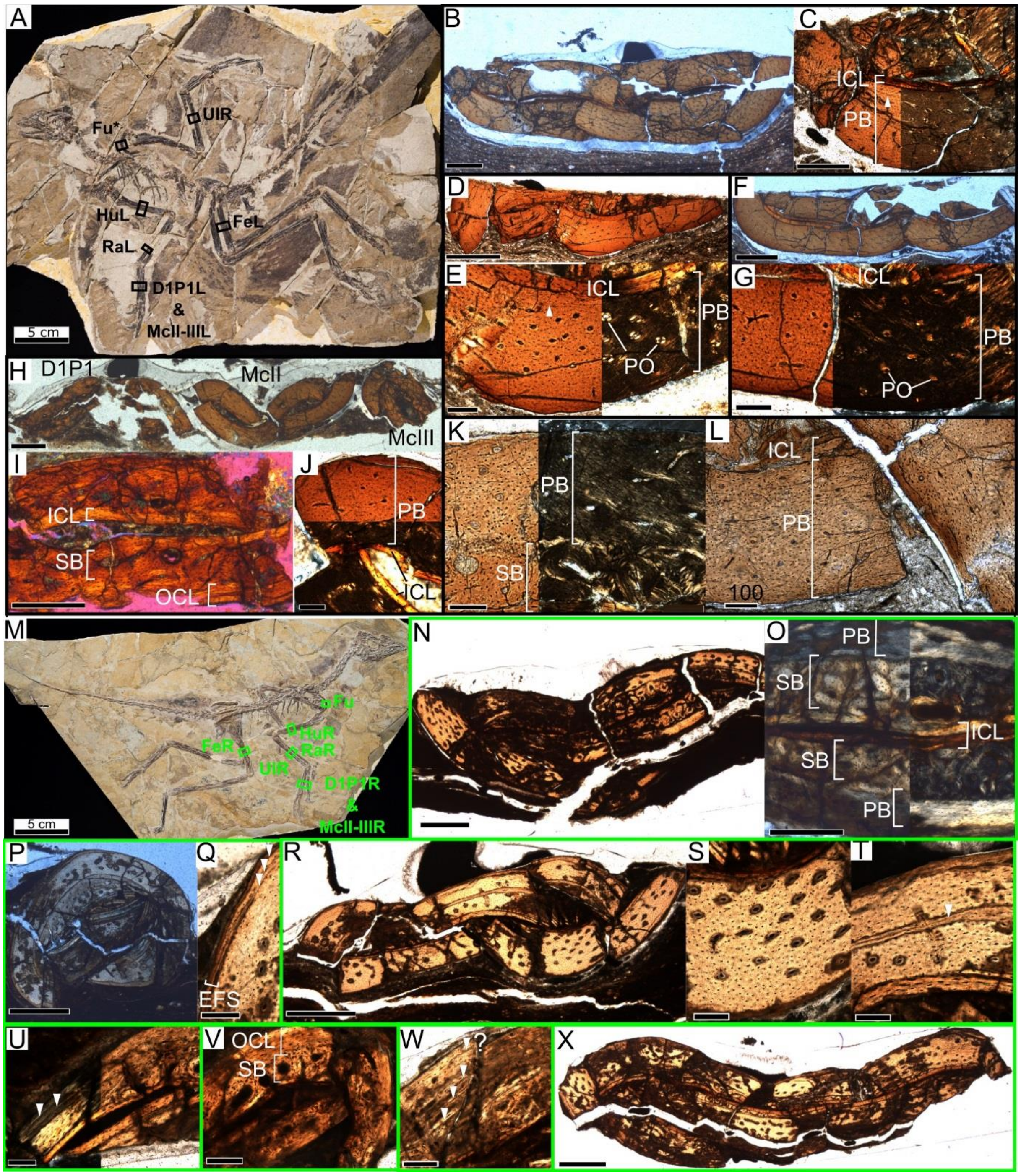

Figure S2. Intraskeletal histodiversity in the subadult *Anchiornis huxleyi* YFGP – T5199 and the adult *Aurornis xui* YFGP – T5198. A, *Anchiornis* YFGP – T5199 skeleton with indication of sampling locations and histological thin sections of its B-C, humerus; D-E, radius; F-G, ulna; H-J, McII-III and D1P1; and K-L, femur. M, *Aurornis* YFGP – T5198 skeleton with indication of sampling locations and histological thin sections of its N-O, humerus; P-Q, radius; R-T, ulna; U, McII; V, McIII; W, D1P1; and X, femur. White arrowheads point to LAGs. Abbreviations as in Figures 1-2 and Figure S1. Colour codes of specimens as in Figure 3. Scale bars: 600 µm in B,D,F,H,N,P,R,X; 300 µm in C,I,O; 100 µm in E,G,J,K,L,Q,S-W.

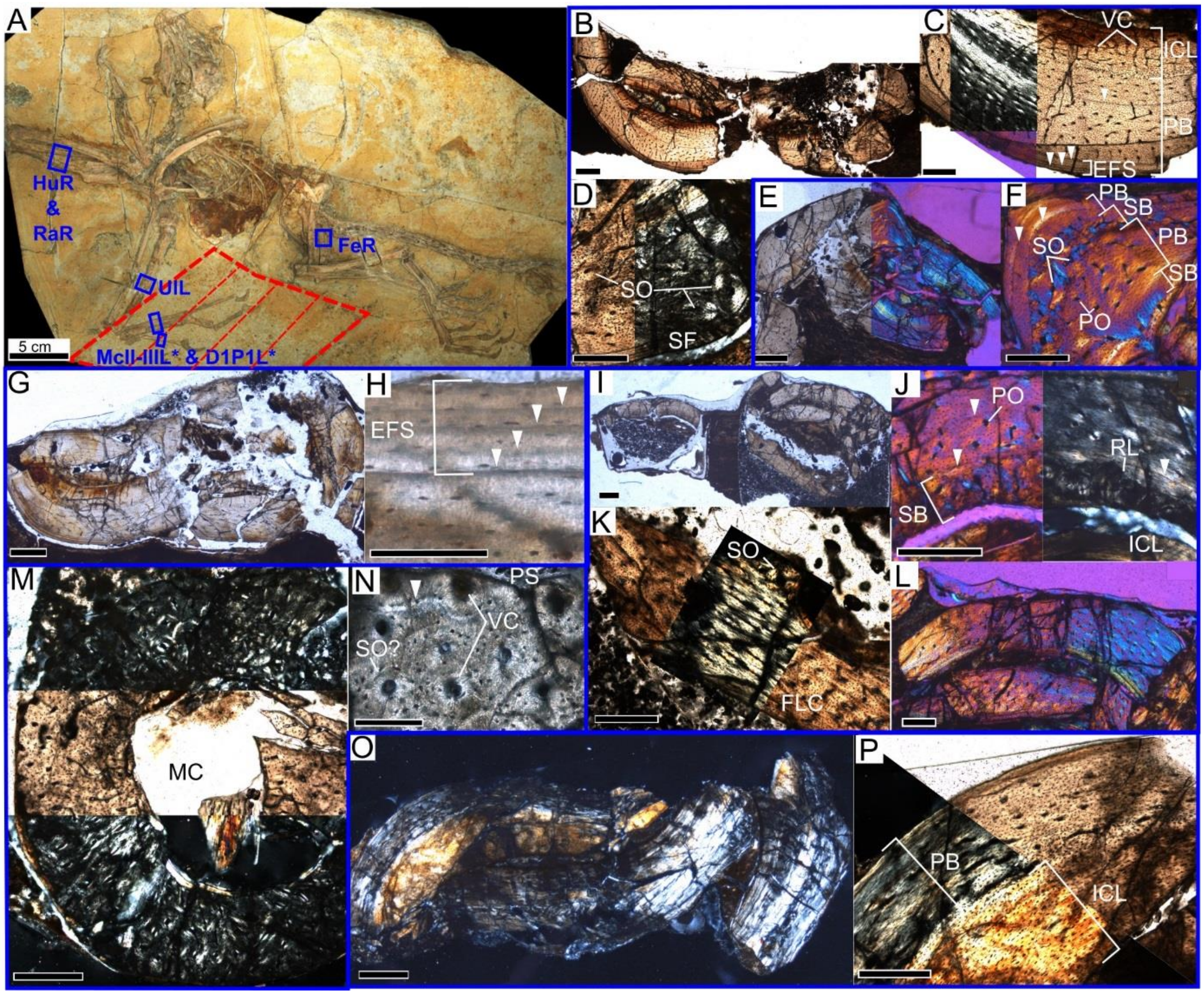

Figure S3. Intraskeletal histodiversity in the adult *Jeholornis curvipes* YFGP – yb2. A, *Jeholornis* YFGP – yb2 skeleton with indication of sampling locations and the area around the left hand that has been restored (red dashed lines). Histological thin sections of B-D, humerus; E-F, radius; G-H, ulna; I-L, 'McII-III'; M-N, 'D1P1'; and O-P, femur. Note the contrasting histological maturity of the left hand bones (I-N) and the rest of the sampled skeletal elements, and the extremely thick and dense ICL in the femur (P). White arrowheads point to LAGs. Abbreviations as in Figures 1-2 and Figure S1. Colour code of specimen as in Figure 3. Scale bars: 600 μm in B,E,G,I,O; 300 μm in C,D,F,J-M,P; 100 μm in H,N.

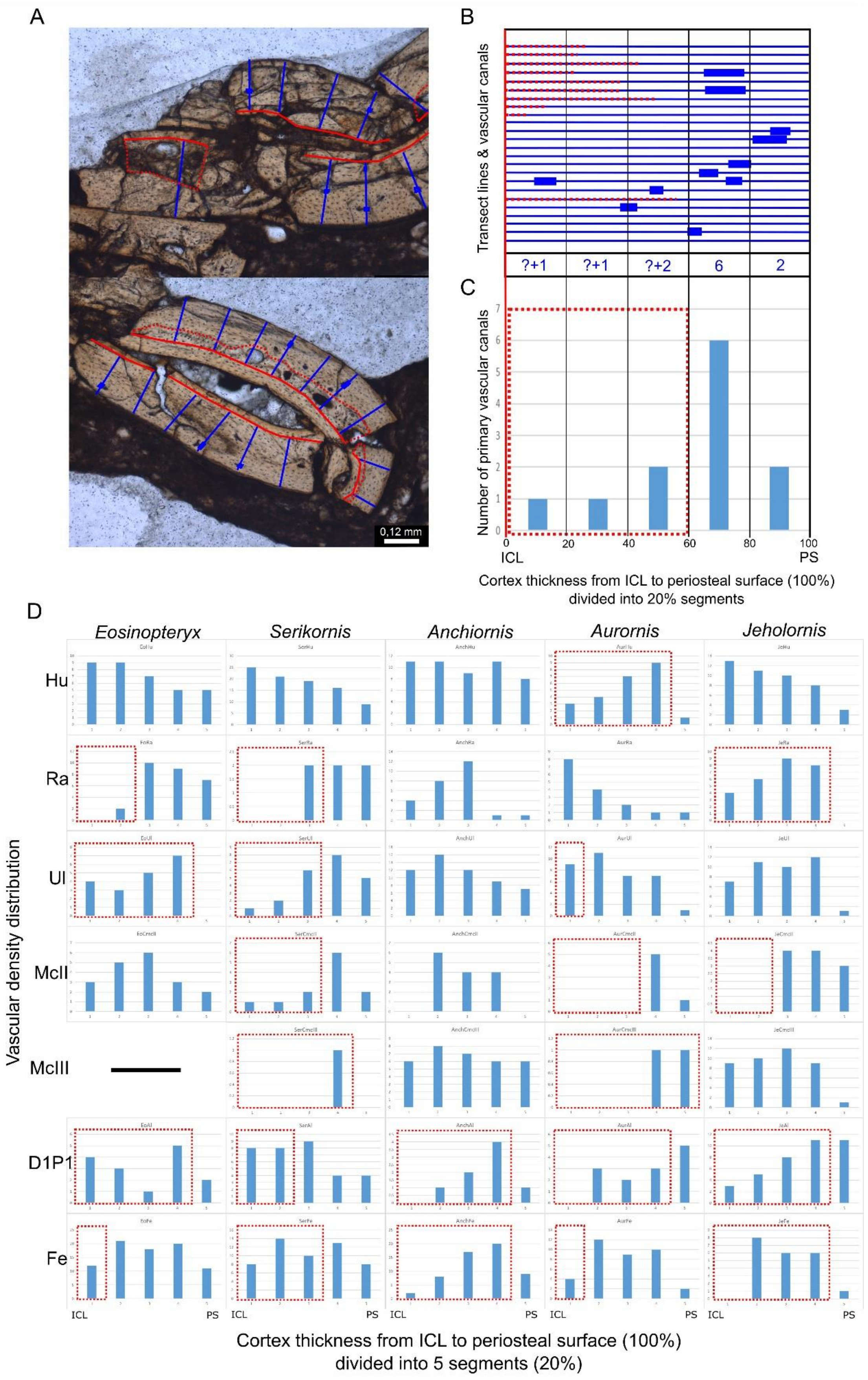
A
B
C
D
0,12 mm
Transect lines & vascular canals
?+1
?+1
?+2
6
2
Number of primary vascular canals
0
20
40
60
80
100
ICL
PS
Cortex thickness from ICL to periosteal surface (100%)
divided into 20% segments
Eosinopteryx
Serikornis
Anchiornis
Aurornis
Jeholornis
Hu
Ra
Ul
McII
McIII
D1P1
Fe
Vascular density distribution
ICL
PS
Cortex thickness from ICL to periosteal surface (100%)
divided into 5 segments (20%)

Figure S4. Measurement technique of radial vascular profiles shown on the example of metacarpal II of *Serikornis sungei* PMOL-AB00200. A, Radial transect lines (blue) running from the resorption line (solid red line) of the inner circumferential layer (ICL) up to the periosteal surface (PS) with the recorded radial extent of primary vascular canals (blue rectangles) that they passed through across the entire preserved cortex. Secondarily remodelled cortical areas are indicated by dotted red line. B, Summarized sequence of adjacent transect lines and the radial extent of vascular canals projected into five equal segments (20%) of the standardized cortex thickness from ICL (0%) up to the periosteal surface (100%). Extent of remodelling on each transect is represented by red dashed lines. Numbers show total number of vascular canals recorded in the corresponding cortical segment. Question marks indicate uncertainty in the maximum number of primary vascular canals along the transects affected by secondary remodelling. C, Overall radial vascular density distribution represented by a histogram with the indication of the maximum recorded radial extent of remodelling (red dotted rectangles) obscuring original primary vasculature. D, Radial vascular density distributions in the sampled bones summarized over all specimens. Abbreviations: D1P1, first phalanx of manual digit I; Fe, femur; Hu, humerus; McII-III, metacarpals II-III, Ra, radius; Ul, ulna.

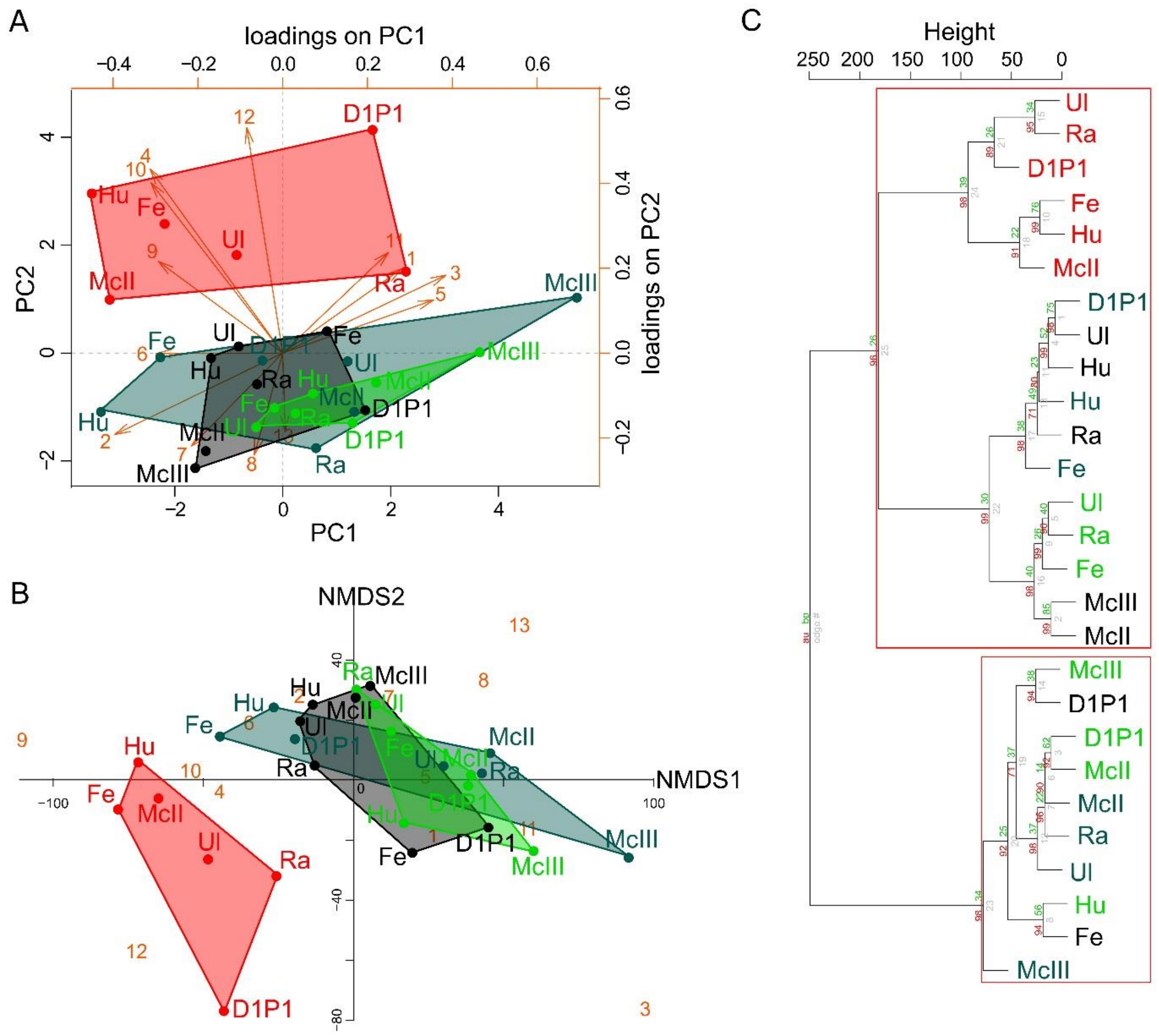

Figure S5. Visual output of different multivariate analyses of histological characters with the inclusion of hand bones but exclusion of *Jeholornis*. A, PC1-PC2 scatterplot with indication of variable loadings (arrows and numbers in orange). B, Two-dimensional NMDS scatterplot with indication of the relative effect of variables on the ordination of elements (labels in orange). Shaded polygons indicate distribution of coherent elements by specimen. C, Cluster dendrogram based on Euclidean distances applying Ward's method with significance values (%) indicated on each cluster branch. Red rectangles mark significant clusters. Note that elements of the juvenile *Eosinopteryx* appear as a distinct group with every method. Colour codes of specimens as in Figure 3. Abbreviation of elements as in Figure S1. Abbreviations of variables as given in Table 3.

Table S1. Length measurements and ratios of individual limb bones of the specimens investigated in this study. Asterisk indicates sampled elements, yellow and green shadings mark the longest and second-longest sampled element of the skeleton, respectively, whereas red cells indicate restored elements. Wherever both right (R) and left (L) sides could be measured, the mean length is given and was used for calculating fore- and hind limb lengths. Forelimb length refers to the additive length of all wing bones down to the distal end of metacarpals II-III (McII-III), whereas hind limb length is the sum of individual hind limb element lengths down to the distal end of the metatarsals (Mt). Forelimb / hind limb ratio (fl/hl) is given accordingly. Abbreviation of elements as in Figure S1.

Element	*Anchiornis*		*Aurornis*		*Eosinopteryx*		*Jeholornis*		*Serikornis*	
	Length (cm)	mean	Length (cm)	mean	Length (cm)	mean	Length (cm)	mean	Length (cm)	mean
Hu R	6.67*		5.93*		3.65*		9.35*		5.67*	
Hu L	6.88	6.77	—		—		—		5.62	5.64
Ra R	5.52*		5*		3.72*		8.65*		5.28	
Ra L	5.31	5.41	—		—		10.21	9.43	5.13*	5.21
Ul R	5.72		5.08*		3.83*		9.52		5.20	
Ul L	5.42*	5.57	—		3.57	3.57	9.07*	9.30	5.22*	5.21
D1P1 R	2.17*		2.4*		1.68		2.90		—	
D1P1 L	—		—		1.48*	1.68	2.92*	2.91	2.59*	
Mc II R	3.09*		3.04*		2.39		4.87		—	
Mc II L	—		—		2.09*	2.24	4.94*	4.91	3.19*	
Mc III R	3.27*		3.19*		2.40		—		—	
Mc III L	3.11	3.19	—		1.96*	2.18	5.25*		2.91*	
Fe L	6.36*		6.02*		3.75		—		7.27	
Fe R	6.68	6.52	—		4.29*	4.02	6.35*		6.81*	7.04
Ti L	9.88		8.91		6.52		8.48		9.45	
Ti R	10.33	10.1	8.10	8.5	6.29	6.41	8.39	8.43	9.35	9.40
Mt L	5.74		3.86		3.35		4.34		4.96	
Mt R	6.04	5.89	3.62	3.74	2.97	3.16	4.44	4.39	4.68	4.82
**forelimb**		15.5		14.2		9.53		23.9		13.8
**hind limb**		22.5		18.3		13.58		19.2		21.3
**ratio (fl/hl)**		0.69		0.78		0.7		1.25		0.65

Background information on quantitative analyses

Histomorphometric measurements were first analysed by basic data exploring methods, such as histograms and Shapiro-Wilk normality test to check distribution attributes of numeric variables, and scatter plots and multivariate pairwise correlation analyses among dependent variables as well as among explanatory variables to assess the type and degree of contribution of highly correlating variables to the results of multivariate analyses.

Thereafter, we applied three different methods to explore and visualize the distribution of skeletal elements in the multivariate histomorphospace: 1) principal component analysis (PCA) on correlation matrix, an eigenvector-based method that works with orthogonal transformation to produce uncorrelated axes of maximum variance in a multivariate dataset; 2) non-metric multidimensional scaling (NMDS), an ordination method based on dissimilarity matrices that iteratively ordinates the most similar data points the closest together in a chosen number of dimensions; and 3) hierarchical cluster analysis applying Ward's agglomerative method that works with distance matrices and links object groups with minimum within-group variance increase in a progressive hierarchical process (e.g. Manly, 2005). As these methods are based on different computational principles, they represent complementary multivariate approaches when applied on the same dataset, and their results can be compared to reveal consistent patterns and most supported groups across these different analytical methods. In all analyses, each skeletal element was handled as an 'independent data point' in order to check whether (and if yes, how much) their histological characteristics adhere to any of their *a priori* known natural groups (i.e. homologous bones and intraindividual elements) or rather reflect the more subjectively assigned categories (ontogenetic stages).

To assess which numeric explanatory variables (element length, sampling location, cortex thickness, vascular canal area, precocity ranks) may be associated with the multivariate patterns, we performed linear (Pearson) and rank-based (Spearman) correlation analyses on one hand with the coefficients of principal components with the highest eigenvalue (explaining highest proportion of variance) acquired by PCA, on the other hand with the NMDS coordinates. Thereafter, if multiple explanatory variables correlated significantly and strongly with the same PCA or NMDS scores, they were tested for their proportional explanatory power by variation partitioning complemented by redundancy analysis and analysis of variance for testing significance of fractions, following the concept presented by Cubo et al. (2008).

For testing the significance of our categorical grouping factors (bone type, taxon, ontogenetic stage), we performed permutation-based multivariate analysis of variance (PERMANOVA) in PAST3 free statistical software.

All the other analyses mentioned above were performed in R 3.3.3 statistical language. The significance level for all tests were set at $\alpha = 0.05$.

For implementing our quantitative analyses in R (3.3.3), we used

- function 'shapiro.test' in the standard R 'stats' package for testing distribution normality
- function 'cor.test' in the standard R 'stats' package for bivariate correlations and function 'rcorr' in package 'Hmisc' (Harrell, 2017) for multivariate pairwise correlations

- function ‘prcomp’ in the standard R ‘stats’ package and functions ‘PCA’ and ‘biplot’ in package ‘ChemometricsWithR’ (Wehrens, 2017) for principal component analyses and their visualizations
- functions ‘pvclust’, ‘plot’ and ‘pvrect’ in package ‘pvclust’ (Suzuki & Shimodaira, 2015) for cluster analyses and their dendrogram visualizations with indication of significant clusters tested by multiscale bootstrap resampling
- functions ‘metaMDS’ and ‘ordiplot’ in package ‘vegan’ (Oksanen et al. 2013) for non-metric multidimensional scaling analyses and their visualizations
- function ‘dunn.test’ in package ‘dunn.test’ (Dinno, 2017) for performing Kruskal-Wallis test with Hochberg p correction
- functions ‘varpart’ and ‘rda’ in package ‘vegan’ (Oksanen et al. 2013) and function ‘anova’ in the standard R ‘stats’ package for variation partitioning

Detailed summary of results of quantitative analyses without hand bones

Correlation between "dependent" variables

EL thickness % & PB area %: Rho=-0.65, p=0.0017
& vsc density: Rho=-0.53, p=0.0160
& EFS thickness %: Rho=0.49, p=0.0298

PB area % & SB area %: Rho=-0.8, p<0.0001
& vsc density: Rho=0.49, p=0.0293

Vsc density & WB %: Rho=0.5, p=0.0235
& EFS thickness %: Rho=-0.65, p=0.0019
& vsc diameter %: Rho=0.71, p=0.0004

Long vsc % & circ vsc %: Rho=-0.5, p=0.0234
& obl vsc %: Rho=-0.64, p=0.0025
& rad vsc %: Rho=-0.58, p=0.0069
& irr vsc %: Rho=-0.73, p=0.0003

Obl vsc % & rad vsc %: Rho=0.57, p=0.0090

Vsc area % & WB %: Rho=0.63, p=0.0030

EFS thickness % & vsc diameter: Rho=-0.77, p<0.0000

PCA

Only elements of *Jeholornis* appear as a separate group in PC1-PC2 projection with no overlap with elements of other taxa, while all the rest appear as a point cloud without any

taxonomic, ontogenetic or element separation. Elements of *Jeholornis* appear separated from the rest on PC1 separately as well.

	PC1	PC2	PC3
Proportion of Variance:	42%	23%	11%

PC1: Loadings about equally distributed in PC1 except for secondary bone % and vascular (vsc) area % which have one order of magnitude less effect than the rest. Vsc orientations have the highests. Vascular features and ICL & EFS thickness % have the highest loadings. Among variables with considerable loading, ICL and EFS thickness % and vsc orientations, except long vsc %, are positively correlated with PC1, as opposed to all other variables.

PC2: Loadings in PC2 are equally distributed with ICL thickness % having the least and vsc area % the most effect. Among variables with considerable loading, EFS thickness %, secondary bone % and long vsc % are negatively correlated with PC2 as opposed to all other variables.

PC3: Primary bone % is dominating and vsc orientation has the least effect. Among variables with considerable loading, Primary bone % and EFS thickness are positively correlated with PC3, as opposed to other variables.

Correlation between explanatory variables

cortex thickness & mean vsc area: Rho=0.76, p<0.0001
& element length: Rho=0.82, p<0.0001

mean vsc area & element length: Rho=0.51, p=0.0223

sampling location & precocity rank: Rho=-0.76, p=0.0001

Correlation between PCs & explanatory variables

PC1 & element length: Rho=0.67, p=0.0006
& cortex thickness: Rho=0.64, p=0.0014

PC2 & precocity rank: Rho=0.55, p=0.0121
& sampling location: Rho=-0.5, p=0.0243

PC3 & precocity rank: Rho=0.43, p=0.0300
& sampling location: Rho=-0.53, p=0.0154

Cluster analysis

Elements of both *Eosinopteryx* and *Jeholornis* appear as separate clades on the dendrogram, while other 'clades' are a mixture concerning taxonomic, overall ontogenetic or element composition. Composition and gross typology of "clades" seems to reflect precocity ranks by clustering elements of similar ranks together.

NMDS

Abstraction to 2 dimensions
Eosinopteryx and *Jeholornis* in 2D MDS1-MDS2 appear as separate groups with no overlap with elements of other taxa / ontogenetic stage, but all the rest appear as a point cloud without any taxonomic, ontogenetic or element separation. Projection on MDS1 shows that only elements of *Jeholornis* but not those of *Eosinopteryx* can be separated from the rest, while on MDS2 none of them can.

Correlation between MDSs & explanatory variables

MDS1 & element length: Rho=0.7, p=0.0003
& cortex thickness: Rho=0.64, p=0.0015

MDS2 & precocity rank: Rho=0.59, p=0.0028
& sampling location: Rho=-0.66, p=0.0008

MDS3 & mean vsc area: Rho=0.49, p=0.0146

Variation partitioning in PCA and MDS scores

PC1:Element length and cortex thickness explain 66% of total variance of which their interaction explains the most and is the only significant fraction.

PC2: Precocity ranks and sampling location explain only 25% of total variance in PC2 scores of which their interaction explains the most and is the only significant fraction.

MDS1: Element length and cortex thickness explain 47% of total variance in MDS1 scores of which their interaction explains the most and is the only significant fraction.

MDS2: Precocity ranks and sampling location explain 39% of total variance in MDS2 scores of which their negative interaction explains the most and is the only significant fraction.

PERMANOVA (performed in PAST3)

Significant results are highlighted by yellow cells. Only the ontogenetic stage has significant grouping effect on the multivariate dataset, and only the juvenile (*Eosinopteryx*) is significantly different from adults.

Grouping factor: Element

Permutation N:	9999
Total sum of squares:	4.91E+07
Within-group sum of squares:	4.61E+07
F:	0.3468
p (same):	0.9324

uncorrected p-values

**Elements**	Hu	Ra	Ul
Hu			
Ra	0.455		
Ul	0.7609	0.8511	
Fe	0.8829	0.7568	0.8544

Grouping factor: Ontogenetic stage

Permutation N:	9999
Total sum of squares:	4.91E+07
Within-group sum of squares:	2.45E+07
F:	5.34
p (same):	0.0009

Bonferroni p-values

**Ont. Stage**	subadult	adult	juvenile
subadult			
adult	0.324		
juvenile	0.1662	0.0132	
late juvenile	1	0.828	0.3318

Grouping factor: Taxon

Permutation N:	9999
Total sum of squares:	4.91E+07
Within-group sum of squares:	1.68E+07
F:	7.207
p (same):	0.0001

Bonferroni p-values

**Taxon**	Anch	Aur	Eos	Jeh
Anch				
Aur	1			
Eos	0.309	0.286		
Jeh	0.275	0.293	0.283	
Ser	1	1	0.547	0.295

Detailed summary of results of quantitative analyses with hand bones but without *Jeholornis*

Correlation between "dependent" variables

EL thickness % & PB area %: Rho=-0.64, p=0.0004
& SB area %: Rho=0.49, p=0.0093
& vsc diameter: Rho=0.58, p=00016

PB area % & SB area %: Rho=-0.95, p=0.0000
& vsc density: Rho=0.41, p=0.0347
& long vsc %: Rho=-0.5, p=0.0077
& irr vsc %: Rho=0.48, p=0.0107
& vsc area %: Rho=0.49, p=0.0103

SB area % & vsc density: Rho=-0.42, p=0.0310
& long vsc %: Rho=0.52, p=0.0051
& circ vsc %: Rho=-0.39, p=0.0455
& irr vsc %: Rho=-0.57, p=0.0020
& vsc area %: Rho=-0.43, p=0.0237

Vsc density & circ vsc %: Rho=0.42, p=0.0279
& irr vsc %: Rho=0.66, p=0.0002
& vsc area %: Rho=0.84, p=0.0000
& WB %: Rho=0.49, p=0.0100

Long vsc % & circ vsc %: Rho=-0.5, p=0.0079
& obl vsc %: Rho=-0.7, p=0.0000
& rad vsc %: Rho=-0.58, p=0.0017
& irr vsc %: Rho=-0.44, p=0.0204

Irr vsc % & vsc area %: Rho=0.55, p=0.0032

Vsc area % & WB %: Rho=0.54, p=0.0034

EFS thickness % & vsc diameter: Rho=-0.56, p=0.0022

PCA

Eosinopteryx in PC1-PC2 2D projection has no overlap with elements of other taxa / ontogenetic stage, but all the rest appear as a point cloud without any taxonomic, ontogenetic or element separation. However, elements of *Eosinopteryx* do not group closely together but are rather spread in a large area in PC1-PC2 and they do not separate on PC1 axis.

	PC1	PC2	PC3
Proportion of Variance:	35%	18%	15%

Loadings are about equally distributed in PC1, except for radial vsc %, woven bone (WB) %, EFS thickness % which have one, one and two orders of magnitude less effect, respectively. Primary and secondary bone % have the highest loading values. ICL and EFS thickness %, secondary bone %, long vsc % and vsc diameter % are positively correlated with PC1 as opposed to all other variables.

Loadings in PC2 show least effect of circular vsc %; the rest are of the same magnitude, although WB % has the highest value. Primary bone %, circ, obl & rad vsc % and EFS thickness % are negatively correlated with PC2 as opposed to all other variables.

PC3 is dominated by EFS thickness % and circ vsc has the least effect. Primary bone %, vsc density, long vsc %, vsc area % and EFS thickness are positively correlated with PC3 as opposed to all other variables.

Correlation between explanatory variables

cortex thickness & mean vsc area: Rho=0.64, p=0.0004
& element length: Rho=0.79, p<0.0001
& precocity rank: Rho=0.63, p=0.0004

element length & precocity rank: Rho=0.54, p=0.0004

sampling location & precocity rank: Rho=-0.45, p=0.0178

Correlation between PCs & explanatory variables

PC1 & precocity rank: Rho=-0.74, p<0.0001
& cortex thickness: Rho=-0.5, p=0.0075
& mean vsc area: Rho=0.52, p=0.0055

PC2 & precocity rank: Rho=0.72, p<0.0001

PC3 & element length: Rho=0.51, p=0.0031
& precocity rank: Rho=0.46, p=0.0075
& cortex thickness: Rho=0.38, p=0.00236

Cluster analysis

Eosinopteryx appears as a separate clade on the dendrogram; however, other 'clades' are a mixture concerning taxonomic, overall ontogenetic or element composition. Composition and internal structure of "clades" more or less conforms to precocity ranks.

NMDS

Abstraction to 3 dimensions
Eosinopteryx in MDS1-MDS2-MDS3 appears as a separate group with no overlap with elements of other taxa / ontogenetic stage, but all the rest appear as a point cloud without any

taxonomic, ontogenetic or element separation. However, projections on neither of these three axes results in separation of *Eosinopteryx* elements.

Correlation between MDS scores & explanatory variables

MDS1 & precocity rank: Rho=-0.49, p=0.0096
& mean vsc area: Rho=0.47, p=0.0130

MDS2 & precocity rank: Rho=0.72, p<0.0001
& cortex thickness: Rho=0.43, p=0.0137
& sampling location: Rho=-0.36, p=0.0179

MDS3 & mean vsc area: Rho=0.45, p=0.0087

Variation partitioning in PCA and MDS scores

PC1: Precocity rank, cortex thickness & mean vsc area explain 49% of total variance in PC1 of which precocity rank explains the majority (pure effect: 23%, together with interactions: 47%)

PC3: Precocity ranks and element length explains only 25% of total variance in PC3 scores of which their interaction explains the most and is the only significant fraction (16%).

MDS1: Precocity ranks and mean vsc area explain only 25% of total variance in MDS1 scores of which mean vsc area explains more but their interaction the most. Only the whole model and the combined explanatory variables are significant explanatory fractions for MDS1 but the overall 25% explanatory power is still very low.

MDS2: Cortex thickness & precocity ranks explain 45% of total variance in MDS2 scores of which precocity rank explains the majority (pure effect: 31%, together with interactions: 47% (due to negative correlation)). Cortex thickness alone is not a significant explanatory fraction for MDS2.

PERMANOVA (performed in PAST3)

Significant results are highlighted by yellow cells. Only *Eosinopteryx* separates from the rest of the specimens both, as a taxonomic and ontogenetic unit.

Grouping factor: Element

Permutation N:	9999
Total sum of squares:	6.68E+07
Within-group sum of squares:	5.44E+07
F:	0.9622
p (same):	0.4744

uncorrected p-values

**Element**	Hu	Ra	Ul	Mc	Al
Hu					
Ra	0.4344				
Ul	0.6603	0.7725			
Mc	0.1319	0.5199	0.3332		
Al	0.2506	0.6249	0.5769	0.4577	
Fe	0.8873	0.5454	0.6884	0.1606	0.5176

Grouping factor: Ontogenetic stage

Permutation N:	9999
Total sum of squares:	6.68E+07
Within-group sum of squares:	3.67E+07
F:	6.296
p (same):	0.0003

Bonferroni p-values

**Ont. stage**	subadult	adult	juvenile
subadult			
adult	0.9282		
juvenile	0.0036	0.0042	
late juvenile	1	1	0.0162

Grouping factor: Taxon

Permutation N:	9999
Total sum of squares:	6.68E+07
Within-group sum of squares:	3.67E+07
F:	6.296
p (same):	0.0003

Bonferroni p-values

**Taxon**	Anch	Aur	Eos
Anch			
Aur	0.9192		
Eos	0.0072	0.0036	
Ser	1	1	0.021

R script of quantitative multivariate analyses

```
data<-read.csv("dinobird_multivar_noHand.csv",row.names=1,header=TRUE,sep=";",dec=".")
str(data)
'data.frame':   20 obs. of  13 variables:
 $ endlamth_prc : num  8.6 12.2 10.6 10.5 10.4 12.7 7.8 10.5 4.4 21.7 ...
 $ prim_area_prc: num  93.4 77.2 88.8 49.8 53.1 ...
 $ sec_area_prc : num  0 13.4 0 38.7 27.3 ...
 $ vsc_dens     : num  87 95 94 71.3 73.7 ...
 $ long_vsc_prc : num  91.9 88 92.2 92.5 92.4 97.6 92 93.1 87.9 98.5 ...
 $ circ_vsc_prc : num  2.9 4 3 4.6 5.3 1.6 1.8 4.2 5 0 ...
 $ obl_vsc_prc  : num  1.7 4 1.2 1.7 0.7 0 0.9 1.9 0.7 0 ...
 $ rad_vsc_prc  : num  1.7 3.2 1.2 0.8 0 0 0.9 0.4 1.8 0 ...
```

```
 $ irr_vsc_prc  : num  1.9 0.8 2.4 0.4 1.5 0.8 4.4 0.4 4.6 1.5 ...
 $ vsc_area_prc : num  3.2 2 2.6 1.9 1.5 1.8 1 2 6 1.9 ...
 $ WB_prc       : num  8.5 3.8 7.5 14.7 3.2 0.9 1.8 8 34.2 5 ...
 $ EFS_th_prc   : num  0 0 0 0 8.7 6.7 6.1 8.2 0 0 ...
 $ vsc_diam_prc : num  2 2.5 2.5 1.8 1.6 1.5 1.7 1.6 3.3 3.2 ...


# Basic Scatterplot Matrix

pairs(~endlamth_prc+prim_area_prc+sec_area_prc+vsc_dens+long_vsc_prc+
circ_vsc_prc+obl_vsc_prc+rad_vsc_prc+irr_vsc_prc+vsc_area_prc+WB_prc+EFS_th_prc+vsc_diam_pr
c,data=data,cex=0.8)

# Pairwise correlations ###
library(Hmisc)
matrix<-as.matrix(data)
multivar_corr<-rcorr(matrix, type="spearman")


### PCA

pca<-prcomp(data,center=TRUE,scale.=TRUE)
pca
summary(pca)

### Plot with loadings (need to standardize data first manually)

library(ChemometricsWithR)
data_std<-scale(data,center = TRUE, scale = TRUE)
PCA<-PCA(data_std)
biplot(PCA,pc=c(1,2),show.names="both",xlab="PC1",ylab="PC2",score.col=1,loading.col="blue",min.
length=.01)
biplot(PCA,pc=c(2,3),show.names="both",xlab="PC2",ylab="PC3",score.col=1,loading.col="blue",min.
length=.01)


### Plotting & correlation between PC scores vs. explanatory variables

PCs<-pca$x[,1:3]
explanatory<-read.csv("explanatory_vars_noHand.csv",row.names=1,header=TRUE,sep=";",dec=".")
explanatory$ont_rank<-factor(explanatory$ont_rank,labels=c("juvenile","late
juvenile","subadult","adult"))
explanatory$cortexth<-as.numeric(explanatory$cortexth)
explanatory$samp_loc<-as.numeric(explanatory$samp_loc)

str(explanatory)
'data.frame':   20 obs. of  8 variables:
 $ cortexth     : num  612 437 392 511 422 456 344 492 414 275 ...
```

```
 $ mean_vsc_area: num  317 238 271 270 145 ...
 $ length_cm    : num  6.7 5.5 5.4 6.4 5.9 5 5.1 6 3.65 3.7 ...
 $ rank         : num  4 1 3 2 1.5 3.5 3.5 1.5 4 1.5 ...
 $ ont_rank     : Factor w/ 4 levels "juvenile","late juvenile",..: 3 3 3 3 4 4 4 4 1 1 ...
 $ taxon        : Factor w/ 5 levels "Anch","Aur","Eos",..: 1 1 1 1 2 2 2 2 3 3 ...
 $ element      : Factor w/ 4 levels "Fe","Hu","Ra",..: 2 3 4 1 2 3 4 1 2 3 ...
 $ samp_loc     : num  52 66 47 60 65 46 51 62 55 64 ...


### Pairwise correlation of explanatory variables

library(Hmisc)
matrix_expl<-as.matrix(explanatory[,c(1:4,8)])
expl_corr<-rcorr(matrix_expl, type="spearman")

PCs_explanatory<-cbind(PCs,explanatory)

### Element length & PCs

plot(PCs_explanatory$length_cm,PCs_explanatory$PC1)
shapiro.test(PCs_explanatory$length_cm)
shapiro.test(PCs_explanatory$PC1)
cor.test(PCs_explanatory$length_cm,PCs_explanatory$PC1,alternative="greater",method="spearma
n")

plot(PCs_explanatory$length_cm,PCs_explanatory$PC2)
shapiro.test(PCs_explanatory$PC2)
cor.test(PCs_explanatory$length_cm,PCs_explanatory$PC2,alternative="two.sided",method="pearso
n")

plot(PCs_explanatory$length_cm,PCs_explanatory$PC3)
shapiro.test(PCs_explanatory$PC3)
cor.test(PCs_explanatory$length_cm,PCs_explanatory$PC3,alternative="two.sided",method="pearso
n")


### Precocity ranks & PCs

plot(PCs_explanatory$rank,PCs_explanatory$PC1)
cor.test(PCs_explanatory$rank,PCs_explanatory$PC1,alternative="two.sided",method="spearman")

plot(PCs_explanatory$rank,PCs_explanatory$PC2)
cor.test(PCs_explanatory$rank,PCs_explanatory$PC2,alternative="two.sided",method="spearman")

plot(PCs_explanatory$rank,PCs_explanatory$PC3)
cor.test(PCs_explanatory$rank,PCs_explanatory$PC3,alternative="greater",method="spearman")
```

```
### Cortex thickness & PCs

plot(PCs_explanatory$cortexth,PCs_explanatory$PC1)
shapiro.test(PCs_explanatory$cortexth)
cor.test(PCs_explanatory$cortexth,PCs_explanatory$PC1,alternative="greater",method="spearman"
)

plot(PCs_explanatory$cortexth,PCs_explanatory$PC2)
cor.test(PCs_explanatory$cortexth,PCs_explanatory$PC2,alternative="two.sided",method="spearma
n")

plot(PCs_explanatory$cortexth,PCs_explanatory$PC3)
cor.test(PCs_explanatory$cortexth,PCs_explanatory$PC3,alternative="two.sided",method="spearma
n")


### Mean vsc area & PCs

plot(PCs_explanatory$mean_vsc_area,PCs_explanatory$PC1)
shapiro.test(PCs_explanatory$mean_vsc_area)
cor.test(PCs_explanatory$mean_vsc_area,PCs_explanatory$PC1,alternative="greater",method="spe
arman")

plot(PCs_explanatory$mean_vsc_area,PCs_explanatory$PC2)
cor.test(PCs_explanatory$mean_vsc_area,PCs_explanatory$PC2,alternative="two.sided",method="s
pearman")

plot(PCs_explanatory$mean_vsc_area,PCs_explanatory$PC3)
cor.test(PCs_explanatory$mean_vsc_area,PCs_explanatory$PC3,alternative="two.sided",method="s
pearman")


## Sampling location & PCs

plot(PCs_explanatory$samp_loc,PCs_explanatory$PC1)
shapiro.test(PCs_explanatory$samp_loc)
cor.test(PCs_explanatory$samp_loc,PCs_explanatory$PC1,alternative="two.sided",method="spearm
an")

plot(PCs_explanatory$samp_loc,PCs_explanatory$PC2)
cor.test(PCs_explanatory$samp_loc,PCs_explanatory$PC2,alternative="two.sided",method="pearso
n")

plot(PCs_explanatory$samp_loc,PCs_explanatory$PC3)
cor.test(PCs_explanatory$samp_loc,PCs_explanatory$PC3,alternative="two.sided",method="pearso
n")
```

```
plot(PCs_explanatory$ont_rank,PCs_explanatory$PC1,xlab="Overall ontogenetic stage",ylab="PC1 scores")
plot(PCs_explanatory$ont_rank,PCs_explanatory$PC2,xlab="Overall ontogenetic stage",ylab="PC2 scores")
plot(PCs_explanatory$ont_rank,PCs_explanatory$PC3,xlab="Overall ontogenetic stage",ylab="PC3 scores")

plot(PCs_explanatory$taxon,PCs_explanatory$PC1,xlab="Taxa",ylab="PC1 scores")
plot(PCs_explanatory$taxon,PCs_explanatory$PC2,xlab="Taxa",ylab="PC2 scores")
plot(PCs_explanatory$taxon,PCs_explanatory$PC3,xlab="Taxa",ylab="PC3 scores")

plot(PCs_explanatory$element,PCs_explanatory$PC1,xlab="Element",ylab="PC1 scores")
plot(PCs_explanatory$element,PCs_explanatory$PC2,xlab="Element",ylab="PC2 scores")
plot(PCs_explanatory$element,PCs_explanatory$PC3,xlab="Element",ylab="PC3 scores")


##### Cluster analysis with p values, transposed dataset needed

library(pvclust)
data_trans<-t(data)
fit<-pvclust(data_trans, method.hclust="ward.D2",method.dist="euclidean")

# dendogram with p values
plot(fit)

# add rectangles around groups highly supported by the data
pvrect(fit, alpha=.95)



#### NMDS with "vegan"

library(vegan)
nmds<-metaMDS(data,distance="euclidean",k=3,trymax=500,autotransform=FALSE,noshare=FALSE)
stressplot(nmds,pch=20)
ordiplot(nmds,type="t")
ordiplot(nmds,choice=c(2,3),type="t")


### Scatter and correlation analysis on MDS scores vs. explanatory variables

nmds_explanatory<-cbind(nmds$points,explanatory)


### Correlation between NMDS scores and explanatory variables

### Element length
```

```
plot(nmds_explanatory$length_cm,nmds_explanatory$MDS1)
shapiro.test(nmds_explanatory$length_cm)
shapiro.test(nmds_explanatory$MDS1)
cor.test(nmds_explanatory$length_cm,nmds_explanatory$MDS1,alternative="greater",method="pearson")

plot(nmds_explanatory$length_cm,nmds_explanatory$MDS2)
shapiro.test(nmds_explanatory$MDS2)
cor.test(nmds_explanatory$length_cm,nmds_explanatory$MDS2,alternative="two.sided",method="pearson")

plot(nmds_explanatory$length_cm,nmds_explanatory$MDS3)
shapiro.test(nmds_explanatory$MDS3)
cor.test(nmds_explanatory$length_cm,nmds_explanatory$MDS3,alternative="two.sided",method="pearson")


#### Precocity ranks

plot(nmds_explanatory$rank,nmds_explanatory$MDS1)
cor.test(nmds_explanatory$rank,nmds_explanatory$MDS1,alternative="two.sided",method="spearman")

plot(nmds_explanatory$rank,nmds_explanatory$MDS2)
cor.test(nmds_explanatory$rank,nmds_explanatory$MDS2,alternative="greater",method="spearman")

plot(nmds_explanatory$rank,nmds_explanatory$MDS3)
cor.test(nmds_explanatory$rank,nmds_explanatory$MDS3,alternative="two.sided",method="spearman")


### Cortex thickness

plot(nmds_explanatory$cortexth,nmds_explanatory$MDS1)
shapiro.test(nmds_explanatory$cortexth)
cor.test(nmds_explanatory$cortexth,nmds_explanatory$MDS1,alternative="greater",method="spearman")

plot(nmds_explanatory$cortexth,nmds_explanatory$MDS2)
cor.test(nmds_explanatory$cortexth,nmds_explanatory$MDS2,alternative="greater",method="spearman")

plot(nmds_explanatory$cortexth,nmds_explanatory$MDS3)
cor.test(nmds_explanatory$cortexth,nmds_explanatory$MDS3,alternative="two.sided",method="spearman")
```

```
### Mean vascular canal area

plot(nmds_explanatory$mean_vsc_area,nmds_explanatory$MDS1)
shapiro.test(nmds_explanatory$mean_vsc_area)
cor.test(nmds_explanatory$mean_vsc_area,nmds_explanatory$MDS1,alternative="two.sided",method="spearman")

plot(nmds_explanatory$mean_vsc_area,nmds_explanatory$MDS2)
cor.test(nmds_explanatory$mean_vsc_area,nmds_explanatory$MDS2,alternative="two.sided",method="spearman")

plot(nmds_explanatory$mean_vsc_area,nmds_explanatory$MDS3)
cor.test(nmds_explanatory$mean_vsc_area,nmds_explanatory$MDS3,alternative="greater",method="spearman")

plot(nmds_explanatory$samp_loc,nmds_explanatory$MDS1)
shapiro.test(nmds_explanatory$samp_loc)
cor.test(nmds_explanatory$samp_loc,nmds_explanatory$MDS1,alternative="two.sided",method="pearson")

plot(nmds_explanatory$samp_loc,nmds_explanatory$MDS2)
cor.test(nmds_explanatory$samp_loc,nmds_explanatory$MDS2,alternative="less",method="pearson")

plot(nmds_explanatory$samp_loc,nmds_explanatory$MDS3)
cor.test(nmds_explanatory$samp_loc,nmds_explanatory$MDS3,alternative="two.sided",method="pearson")

plot(nmds_explanatory$ont_rank,nmds_explanatory$MDS1,xlab="Overall ontogenetic stage",ylab="NMDS1 scores")
plot(nmds_explanatory$ont_rank,nmds_explanatory$MDS2,xlab="Overall ontogenetic stage",ylab="NMDS2 scores")
plot(nmds_explanatory$ont_rank,nmds_explanatory$MDS3,xlab="Overall ontogenetic stage",ylab="NMDS3 scores")


plot(nmds_explanatory$taxon,nmds_explanatory$MDS1,xlab="Taxa",ylab="NMDS1 scores")
plot(nmds_explanatory$taxon,nmds_explanatory$MDS2,xlab="Taxa",ylab="NMDS2 scores")
plot(nmds_explanatory$taxon,nmds_explanatory$MDS3,xlab="Taxa",ylab="NMDS3 scores")

plot(nmds_explanatory$element,nmds_explanatory$MDS1,xlab="Element",ylab="NMDS1 scores")
plot(nmds_explanatory$element,nmds_explanatory$MDS2,xlab="Element",ylab="NMDS2 scores")
plot(nmds_explanatory$element,nmds_explanatory$MDS3,xlab="Element",ylab="NMDS3 scores")


### Boxplots among explanatory variables
```

```
plot(explanatory$ont_rank,explanatory$mean_vsc_area, xlab="Overall ontogenetic stage",ylab="mean vascular area")
plot(explanatory$ont_rank,explanatory$cortexth,xlab="Overall ontogenetic stage",ylab="cortex thickness")
plot(explanatory$taxon,explanatory$mean_vsc_area,xlab="Taxa",ylab="mean vascular area")
plot(explanatory$taxon,explanatory$cortexth,xlab="Taxa",ylab="cortex thickness")
plot(explanatory$element,explanatory$mean_vsc_area,xlab="element",ylab="mean vascular area")
plot(explanatory$element,explanatory$cortexth,xlab="element",ylab="cortex thickness")
plot(explanatory$element,explanatory$rank,xlab="element",ylab="precocity rank")


## Element vs precocity rank

### Kruskal-Wallis test with Hochberg p correction instead of ANOVA

library(dunn.test)
dunn.test(explanatory$rank,explanatory$element,method="hochberg")
----------------------------------------

### Variation partitioning for decoupling element length, precocity ranks, sampling location, cortex thickness & mean vsc area in explaining PC & MDS scores on the example of PC1

library(vegan)
PC1<-pca$x[,1]
VP_PC1<-varpart(PC1,explanatory$length_cm,explanatory$cortexth,data=explanatory)
VP_PC1
plot(VP_PC1)
## fraction (a+b+c)
RDA_PC1<-rda(PC1~explanatory$length_cm+explanatory$cortexth,data=explanatory)
## fraction (a)
RDA_PC1_a<-rda(PC1~explanatory$length_cm+Condition(explanatory$cortexth),data=explanatory)
## fraction (c)
RDA_PC1_c<-rda(PC1~explanatory$cortexth+Condition(explanatory$length_cm),data=explanatory)
## fraction(a+b)
RDA_PC1_ab<-rda(PC1~explanatory$length_cm,data=explanatory)
## fraction (b+c)
RDA_PC1_bc<-rda(PC1~explanatory$cortexth,data=explanatory)

## Testing fraction (a+b)
anova(RDA_PC1_ab)

## Testing fraction (b+c)
anova(RDA_PC1_bc)

## Testing fraction (a+b+c; i.e. whole model)
anova(RDA_PC1)

## Testing fraction (a)
```

```
anova(RDA_PC1_a)

## Testing fraction (c)
anova(RDA_PC1_c)
```

References

Cubo, J., Legendre, P., de Ricqlès, A., Montes, L., de Margerie, E., Castanet, J. & Desdevises, Y. Phylogenetic, functional, and structural components of variation in bone growth rate of amniotes. *Evol. Dev.* **10**, 217–227 (2008).

Dinno, A. Package ‘dunn.test’. Dunn's Test of Multiple Comparisons Using Rank Sums. (2017) https://cran.r-project.org/web/packages/dunn.test/dunn.test.pdf

Harrell, F.E. Package ‘Hmisc’. Harrell Miscellaneous. (2017) https://cran.r-project.org/web/packages/Hmisc/Hmisc.pdf

Manly, B.F.J. *Multivariate Statistical Methods: A Primer*. 3rd edition. (Chapman & Hall/CRC Press, 2005).

Oksanen, J. *et al*. Package ‘vegan’. Community Ecology Package. (2013) http://cran.r-project.org, http://vegan.r-forge.r-project.org/

Suzuki, R. & Shimodaira, H. package ‘pvclust’. Hierarchical Clustering with P-Values via Multiscale Bootstrap Resampling. (2015) http://www.sigmath.es.osaka-u.ac.jp/shimo-lab/prog/pvclust/

Wehrens, R. Package ‘ChemometricsWithR’. Chemometrics with R - Multivariate Data Analysis in the Natural Sciences and Life Sciences. (2017) https://cran.r-project.org/.../ChemometricsWithR/ChemometricsWithR
